# Factors associated with farmers’ well-being in meaningful environments: a chain psychological association of digital literacy, psychological empowerment, and farmers’ decision-making behaviors

**DOI:** 10.3389/fpsyg.2026.1891582

**Published:** 2026-07-28

**Authors:** Meng Luo

**Affiliations:** Business School, Xi’an Innovation College of Yan’an University, Xi’an, China

**Keywords:** chain mediation, digital literacy, farmers’ decision-making behaviors, farmers’ sense of happiness, meaningful environmental perception, psychological empowerment

## Abstract

**Introduction:**

The formation of farmers’ well-being is closely related to digital literacy, psychological empowerment, and agricultural planting decision-making behavior. In order to explore the underlying sequential associations regarding farmers’ well-being in meaningful environments, this study aims to examine these interrelationships drawing upon positive psychology theories, such as meaning construction theory.

**Methods:**

A chain mediation model was constructed to assess the sequential correlations among the variables. The reliability and validity of the measurement scales were evaluated using Cronbach’s *α*, the KMO measure, and structural fit indices. Multicollinearity among predictor variables was checked using the Variance Inflation Factor (VIF), and the sequential associations were analyzed utilizing a Bootstrap chain mediation procedure.

**Results:**

The analysis indicated that the measurement scales possessed good structural validity and reliability (Cronbach’s α range: 0.870 ~ 0.925; KMO = 0.978; Bartlett’s test *χ*^2^ = 18135.643, *df* = 595, *p* < 0.001; CFI = 0.997, TLI = 0.997, RMSEA = 0.004, SRMR = 0.020). No severe multicollinearity was observed (VIF range: 1.888 ~ 2.105). Meaningful environmental perception was significantly and positively correlated with farmers’ well-being (*r* = 0.616). In the Bootstrap analysis, the total statistical effect of meaningful environmental perception on well-being was 0.6280.628 (direct effect = 0.203; indirect effect = 0.425, accounting for 67.68% of the total effect). The full-chain indirect path—from meaningful environmental perception through digital literacy, psychological empowerment, and farmers’ decision-making behavior to farmers’ well-being—yielded a significant sequential association with a statistical indirect effect of 0.020 (95% CI [0.013, 0.029]).

**Discussion:**

The findings indicate that there are significant positive correlations and sequential indirect associations among meaningful environmental perception, digital literacy, psychological empowerment, decision-making behavior, and well-being in farmers. The chain mediation model offers a relational perspective on how these psychosocial and behavioral factors are intertwined.

## 1 Introduction

Well-being is an important indicator of an individual’s mental health, quality of life, and social adaptation. During rural social transformation, farmers’ happiness is influenced not only by objective factors such as household income, public services, infrastructure, and social security, but also closely related to individuals’ understanding of the significance of the rural environment, confidence in future development, and initiative in agricultural planting (Ma et al., 2024). As digital literacy continues to improve, the internet, smartphones, agricultural information platforms, short video platforms, and e-commerce channels are gradually entering farmers’ daily lives, providing new possibilities for farmers to access information, expand markets, learn technology, and optimize business decisions (Sabillón et al., 2022). However, digital technology itself does not automatically equate to happiness; its role is often systematically associated with continuous transformation through cognitive abilities, psychological resources, and behavioral practice. “Meaningful environments” emphasize that the environment in which individuals exist can provide a sense of value, belonging, purpose, and development possibilities (Knight et al., 2009; Knight and Gunatilaka, 2010). For farmers, the countryside is not only a living space but also an important field for labor practice, family continuity, social relationships, and identity recognition. When farmers see agricultural production as not just a means of livelihood but with family, community, and social significance, they are more likely to gain a lasting sense of meaning from daily labor and rural environments (Burke and Corrigan, 2024). In the context of rural revitalization and digital transformation, improving farmers’ happiness has become not only a livelihood issue but also an important task for promoting sustainable rural development. Although rural infrastructure, public services, household income, and social security have improved in many regions, some farmers still face practical pressures such as unstable agricultural returns, market uncertainty, insufficient digital adaptability, and weak participation in modern agricultural decision-making. These problems indicate that farmers’ happiness cannot be fully explained by objective material conditions alone. More attention should be paid to how farmers understand the value of rural life, whether they perceive development opportunities in the rural environment, and whether they have the ability and confidence to utilize environmental resources for actual well-being. At the same time, when village public services, neighbor relations, digital environments, and industrial development conditions can support farmers in achieving better lives, they are more likely to develop positive psychological expectations and willingness to act. Therefore, meaningful environmental perception may be an important psychological foundation for predicting farmers’ happiness (Cheng and Zhang, 2022). Existing research mainly explores farmers’ well-being from the perspectives of income growth, public service provision, social capital, and rural governance. These studies provide important explanations for the external conditions of farmers’ well-being. However, the psychological mechanisms by which rural environments are associated with happiness have not been sufficiently discussed. Especially in the context of rural revitalization and digital agriculture, farmers are no longer passive recipients of policy support or environmental changes. They are also active participants in acquiring digital information, making production decisions, adjusting business strategies, and building meaningful rural life. Therefore, it is necessary to examine farmers’ sense of well-being from the perspective of “meaningful environmental perception,” that is, their perception of value, belonging, purpose, and future development possibilities in the rural environment. Digital literacy is an important condition for farmers to adapt to improved digital literacy. High digital literacy means farmers can skillfully use smartphones, WeChat, short video platforms, or agricultural information platforms, search for information on agricultural production, market prices, and policy subsidies via the internet, assess the reliability of online agricultural information, and apply it to agricultural planting (Davis, 1989; McCormack et al., 2021). Digital literacy not only improves farmers’ ability to access information but may also enhance their sense of control over production and daily life and confidence in future development. Psychological empowerment reflects individuals’ positive evaluations of their own competence, autonomy, impact, and potential for future change (Cui et al., 2022). In agricultural production scenarios, farmers with higher psychological empowerment believe they can improve their household operations, are more willing to face market fluctuations and agricultural risks, and are more likely to try new technologies, new channels, and new business models. Psychological empowerment further relates to farmers’ decision-making behavior, predicting them to be more proactive and rational in production arrangements, technology adoption, sales channel selection, and risk control (Spreitzer, 1995; Thomas and Velthouse, 1990). Therefore, reasonable decision-making by farmers may be linked to a stronger sense of happiness by improving their living conditions.

Based on the above analysis, this study focuses on farmers and constructs a chain-mediated model for meaningful environmental perception affecting their happiness, focusing on the continuous mechanisms of digital literacy, psychological empowerment, and farmers’ decision-making behaviors. The marginal contributions of this study can be further clarified in three respects. This study extends the research perspective of farmers’ happiness from external material conditions to the psychological meaning of the rural environment. Previous studies have mostly explained farmers’ well-being from the perspectives of income, infrastructure, public services, and social security. This study reveals the associative path from environmental perception to happiness in the context of digital rural development. Existing studies have often discussed digital literacy as a technical ability or information acquisition capacity, but have paid relatively limited attention to how digital literacy is constructively related to psychological resources and behavioral outcomes. This study enriches the practical interpretation of rural revitalization from the perspective of psychological and behavioral intervention. The findings suggest that efforts aiming at improving farmers’ happiness should not rely solely on increasing material investment or improving infrastructure. It is also necessary to enhance farmers’ perception of rural meaning, strengthen their digital literacy, improve their psychological empowerment, and support their decision-making capacity in agricultural production and household management.

## 2 Theoretical foundations and research hypotheses

### 2.1 Meaningful environmental perception and farmers’ sense of well-being

In this study, meaningful environmental perception refers to farmers’ perception of the meaning, value, belonging, and future relevance of the rural environment. Meaningful environmental perception refers to the subjective experience in which individuals believe their living environment can provide value, belonging, purpose, and developmental support (Whitaker, 2024). It is related to sense of place, place attachment, and community satisfaction, but it is not the same concept. Sense of place and place attachment mainly emphasize emotional and cognitive bonds with a place, while community satisfaction focuses on evaluations of local services and living conditions. In contrast, meaningful environmental perception emphasizes whether farmers regard the rural environment as valuable and supportive of their life goals, agricultural participation, and future expectations. Crucially, to distinguish it from the ultimate subjective well-being construct, meaningful environmental perception operates as an outward-directed cognitive appraisal of external physical, social, and structural stimuli in the agricultural context. Subjective well-being, conversely, functions as an inward-directed, comprehensive emotional state reflecting long-term cumulative life satisfaction. In rural settings, farmers’ positive evaluations of village life, agricultural production, neighborly relations, public services, and future development opportunities can enhance their sense of stability in life, social belonging, and hope for the future (Park, 2010). When farmers perceive the rural environment as not only economically functional but also meaningful in terms of family continuity, community belonging, and social relationships, they may be more likely to experience positive emotions and greater life satisfaction in rural life (Seo, 2023).

*H1*: Meaningful environmental perception is positively associated with farmers’ happiness.

### 2.2 The mediating role of digital literacy

Digital literacy refers to farmers’ comprehensive ability to acquire, understand, evaluate, and apply information in a digital rural environment. Farmers with higher levels of digital literacy are more likely to access diverse information beyond traditional sources, including agricultural technologies, market prices, policy subsidies, and sales channels (Li J. et al., 2025; Li R. et al., 2025). Greater information accessibility may be associated with lower perceived uncertainty, a better understanding of the agricultural environment, and more positive expectations regarding future life. Therefore, meaningful environmental perception may be indirectly associated with farmers’ well-being through its positive relationship with digital literacy.

*H2*: Digital literacy plays an independent mediating role between meaningful environmental perception and farmers’ sense of well-being.

### 2.3 The mediating role of psychological empowerment

Psychological empowerment emphasizes an individual’s perceived competence, autonomy, impact, and sense of meaning during the process of action. When farmers have access to effective information and develop a clearer understanding of the practical value of digital tools, they may report a stronger perception of their own capabilities and decision-making autonomy (Deci and Ryan, 2000). The association between digital literacy and farmers’ problem-solving abilities also strengthens their sense of psychological control when facing agricultural risks and market changes (Bandura, 1997; Zimmerman, 1995). The higher the level of psychological empowerment, the more likely farmers are to develop positive emotions and future confidence, which is closely linked to an elevated level of their sense of happiness (Ryff and Keyes, 1995; Spreitzer, 1995).

*H3*: Psychological empowerment plays a mediating role between digital literacy and farmers’ happiness.

### 2.4 The mediating role of farmers’ decision-making behavior

Farmers’ decision-making behavior may serve as an important behavioral pathway through which psychological resources are associated with real-life outcomes. Farmers with higher levels of psychological empowerment are more likely to actively collect information, compare alternative solutions, adopt new technologies, adjust production structures, and participate in cooperative or e-commerce platforms (Thomas and Velthouse, 1990). Such active decision-making behavior may be positively associated with farmers’ perceived control over agricultural production, as well as their subjective perceptions of income prospects, risk response capacity, and quality of life. Therefore, farmers’ decision-making behavior may play an important mediating role in the relationship between psychological empowerment and happiness.

*H4*: Farmers’ decision-making behavior plays a mediating role between psychological empowerment and their sense of happiness.

### 2.5 Chain mediation effect

From an overall mechanism perspective, meaningful environmental perception may be associated with farmers’ positive expectations regarding the future development of rural areas, which may further impact their willingness to learn and use digital tools. Higher levels of digital literacy may also be linked to stronger information acquisition and problem-solving abilities, and these abilities may correspond to a higher level of psychological empowerment. Psychological empowerment further facilitates favorable conditions for farmers to adopt more proactive, rational, and adaptive decision-making behaviors; Digital literacy serves as an important capability bridge between environmental perception and psychological empowerment. Farmers with higher digital literacy are better able to use smartphones, online platforms, agricultural information systems, and e-commerce channels to obtain market information, policy support, technical knowledge, and sales opportunities. Psychological empowerment may encourage farmers to adopt more proactive, rational, and adaptive decision-making behaviors. When farmers perceive greater competence, autonomy, and control over production and livelihood choices, they may become more willing to evaluate alternatives, manage risks, and participate actively in agricultural decision-making. Such participation and capability development may also be associated with higher subjective well-being, although the relationship can depend on workload and contextual constraints (Barrios et al., 2025). From this, it is inferred that there is a positive, continuous intermediary path chain between digital literacy, psychological empowerment, and farmers’ decision-making behavior (Li et al., 2025).

Since digital literacy may have two different paths between meaningful environmental perception and farmers’ well-being, First, the relatively direct path of information capability. Digital literacy can help farmers access and identify market and policy information, reduce perception of uncertainty, and improve the convenience for farmers in handling agricultural production issues. Therefore, digital literacy may directly improve farmers’ living conditions and may act as an independent mediator between meaningful environmental perception and well-being. At the same time, there is also a second possibility: a chain path gradually transitioning from cognitive abilities to behavioral responses. Once farmers possess high digital literacy, they may develop a higher level of competence and autonomy, that is, enhanced psychological empowerment. Then, psychological empowerment may further enhance farmers’ decision-making behaviors in market analysis and risk assessment, ultimately linking psychological empowerment and decision-making behavior to their sense of happiness. Therefore, digital literacy can both act as an independent intermediary and become the starting point of the chain mechanism of “digital literacy—psychological empowerment—farmers’ decision-making behavior.” Therefore, this paper proposes the following hypotheses.

*H5*: Meaningful environmental perception may be linked to farmers’ well-being through a chain mediation of digital literacy, psychological empowerment, and farmers’ decision-making behaviors.

This study sequentially incorporates digital literacy, psychological empowerment, and farmers’ decision-making behaviors into a chain-mediated model, mainly based on the logic of “capability foundation—psychological cognition—behavioral response.” First, digital literacy reflects farmers’ ability to acquire, understand, filter, and use digital information, which is a fundamental ability for individuals to cope with the digital environment. Higher digital literacy may help farmers access more comprehensive market, policy, and production information, reduce information uncertainty, and enhance their understanding and control over the external environment. Second, psychological empowerment reflects an individual’s sense of competence, autonomy, relate to, and control. Digital information capability itself does not necessarily serve as an immediate predictor for actual behavior; only when farmers can further utilize their information capacity to build positive awareness of their own abilities and decision-making control can digital literacy have a more stable impact on behavioral choices. Therefore, digital literacy is placed before psychological empowerment. Finally, farmers’ decision-making behaviors are relatively explicit outcomes, manifested in information comparison, risk assessment, market analysis, and production and operation choices. According to social cognitive theory, individuals’ judgments about their own abilities and levels of control may relate to their behavioral engagement and decision-making tendencies. Therefore, this study places psychological empowerment before decision-making behavior, forming a progressive relationship of “digital literacy—psychological empowerment—farmers’ decision-making behavior.” It should be noted that the above arrangements are mainly based on theoretical deduction and do not mean there is no feedback relationship between variables. For example, positive decision-making outcomes may also enhance farmers’ psychological empowerment and encourage them to continue learning and using digital tools. Therefore, this study regards this chain structure as a theoretically reasonable explanatory model, rather than a confirmation of the sole causal order among variables, necessitating that future studies statistically test potential alternative competitive models to consolidate this structural pathway.

## 3 Research methodology

### 3.1 Research subjects and data sources

This study used questionnaires to collect data (the complete questionnaire is available in Supplementary Material 1). The questionnaire was designed around meaningful environmental perception, digital literacy, psychological empowerment, farmers’ decision-making behaviors and well-being, as well as demographic variables such as gender, age, education level, annual household income, years of agricultural production, type of agricultural operation, and frequency of digital platform use. The survey was conducted from June to October 2025. To improve sample representativeness and data stability, cities with large agricultural planting areas were selected. Before the survey, community directors were contacted to obtain lists of community members, and questionnaires were distributed using a stratified quota-based convenience sampling method. To prevent sampling skewness and match Shaanxi agricultural census profiles, strict quota ceilings for key demographic and operational options were pre-configured in the online survey administration platform. Under this stratified quota system, farmers were selected from the lists provided by community directors. However, the sampling procedure may still involve selection bias. Farmers who were active in community affairs, willing to participate in surveys, or familiar with digital technologies may have been more likely to be included in the available lists and to complete the questionnaire. Therefore, the sample may not fully represent farmers with limited community participation or low digital access. When community directors provide the list of personnel, they classify based on land planting area, planting method (leased, owned), and other types to improve the rationality of the questionnaire structure.

To assure a comprehensive representation, the target farmers were stratified into different subgroups based on age, education level, annual household income, type of agricultural operation, and frequency of digital platform use. This stratification helps ensure that farmers with different demographic characteristics, economic conditions, production patterns, and levels of digital participation are well represented in the sample. In this survey, a total of 900 questionnaires were distributed, 862 were returned, resulting in a response rate of 95.78%. During the data organization stage, 37 invalid questionnaires with short filling times, strong response patterns, missing many key variables, and obvious contradictions were removed. In total, 825 valid questionnaires were collected, with an effectiveness rate of 95.71%. Among them, Weinan City had the most valid questionnaires, with 88 responses (10.67%); Xianyang City and Yulin City each had 87 responses, each accounting for 10.55%; Ankang City and Shangluo City each account for about 10.06%; Xi’an, Baoji, and Hanzhong each accounted for 9.94%; Yan’an City accounts for 9.45%; Tongchuan City accounted for 8.85%.

### 3.2 Research tools

All variables in this study were evaluated using a 5-point Likert scale: 1 indicates “strongly disagree,” 5 means “strongly agree,” with higher scores indicating higher levels of related variables. The questionnaire is divided into two parts: basic information and core variable scales, comprising 35 items in total (the complete questionnaire is available in Supplementary Material 1). Core variables include meaningful environmental perception, digital literacy, psychological empowerment, farmers’ decision-making behavior, and well-being.

The Digital Literacy Scale consists of seven items and was revised with reference to research related to digital literacy and farmers’ production and decision-making (adapted from McCormack et al., 2021). A sample item is DL1: “I am proficient in using smartphones, WeChat, short video platforms, or agricultural information platforms.” Existing studies have shown that digital literacy can enhance farmers’ ability to access information and is positively associated with their production and decision-making behaviors (Gong et al., 2024; Lu et al., 2024).

The Psychological Empowerment Scale contains seven items and is mainly designed based on psychological empowerment theory, emphasizing individuals’ sense of control and agency in social participation and behavioral choices (Perkins and Zimmerman, 1995; Zimmerman, 1995). A sample item is PE1: “I believe I have the ability to improve the family’s agricultural operations.”

The Farmers’ Decision-Making Behavior Scale comprises seven items and has been revised, referencing farmers’ decision-making behaviors, adoption of agricultural technologies, risk tolerance, and research related to rural e-commerce operations. It mainly measures farmers’ tendencies in market research, information gathering, risk control, technology adoption, and participation in e-commerce platforms (Jussaume et al., 2022; Qiu et al., 2025). A sample item is DB1: “Before making agricultural production decisions, I proactively gather market, technical, or policy information.” Existing research shows that farmers’ decision-making processes are related to risk attitudes, market information, technological conditions, and the business environment. This study defines it as a reflective concept, used to reflect the common latent tendencies of farmers to be proactive, rational, information-driven, and risk-aware in agricultural production and management. Drawing on Social Cognitive Theory (Bandura, 1997) and cognitive empowerment theory (Spreitzer, 1995; Thomas and Velthouse, 1990), this framework suggests that cognitive self-beliefs (such as competence, autonomy, and impact) function as intrinsic motivational drivers that shape farmers’ proactive, rational, and adaptive behavioral choices in daily farming operations. Although each item involves different decision-making scenarios, they all reflect the characteristics of farmers using information, comparing plans, assessing risks, and making adaptive decisions.

The happiness (well-being) scale consists of eight items and was revised with reference to subjective happiness and life satisfaction scales, focusing on measuring farmers’ subjective evaluations of living conditions, family safety, future confidence, and well-being. The Life Satisfaction Scale developed by Diener et al. (1985) is mainly used to measure individuals’ cognitive evaluation of overall quality of life, and has good internal consistency and temporal stability, making it suitable as an important theoretical source for well-being measurement. Selected items were also adapted from the psychological well-being framework (Ryff and Keyes, 1995) to capture environmental mastery and personal growth. A sample item is Y1: “Overall, I am satisfied with my current life.”

The Meaningful Environmental Perception Scale is based on the theory of meaning construction and related literature. To build a robust construct, the items were developed and adapted by integrating structural dimensions from three classic sociological and psychological instruments: the Place Attachment Scale (Williams and Vaske, 2003) for environmental belonging, the Sense of Community Scale (McMillan and Chavis, 1986) for neighborhood support and local agency, and the Meaning in Life Questionnaire (Steger et al., 2006) adapted to capture the intrinsic, vocational meaning of agricultural labor. Ultimately, the scale consists of six items: (X1) “I believe the current rural living environment makes me feel valued and a sense of belonging”; (X2) “I can feel that agricultural production is not only a means of livelihood but also carries family, community, or social significance”; (X3) “Public services, neighborhood relations, and production conditions in the village support me in achieving a better life”; and (X4) “I believe I can play a certain role in rural development”; (X5) “The current rural digitalization, industrialization, and governance environment give me greater confidence in future life”; and (X6) “I was able to gain a lasting sense of meaning from agricultural labor and rural life.” Although both constructs relate to positive appraisals in rural life, they capture distinct domains: meaningful environmental perception operates as an environment-oriented evaluation of external attributes (such as belongingness and rural development values), whereas well-being functions as an individual-oriented psychological outcome (such as life satisfaction and future confidence).

The above survey scales were revised based on farmers’ daily lives, agricultural participation, and future development expectations. To ensure measurement equivalence across languages, a double-translation and back-translation procedure was implemented. To enhance content validity, this study invited three experts in rural sociology and psychology to evaluate the expression of the items, concept fit, and contextual applicability. Pre-tests were conducted before the formal survey, and questions with unclear or repetitive expressions were modified, ultimately forming the survey scale of this study.

### 3.3 Data analysis methods

This study analyzed the data using descriptive statistics, reliability analysis, validity analysis, common methods bias testing, Pearson correlation analysis, collinearity analysis, and chain-mediated effect testing. First, frequency and percentage statistics were performed on demographic variables to describe the basic structure of the sample. Subsequently, factor loading, Cronbach’s *α*, combined reliability (CR), and average variance extracted (AVE) were evaluated to test the reliability and validity of the measurement scales. KMO test and Bartlett’s test of sphericity were also used alongside Confirmatory Factor Analysis (CFA) to evaluate overall measurement properties. To robustly verify discriminant validity and address potential conceptual overlaps, the Heterotrait-Monotrait Ratio (HTMT) criterion was supplementary employed alongside the Fornell-Larcker matrix (Henseler et al., 2015). Confirmatory factor analysis was used to evaluate the measurement properties of the scales, whereas the chain mediation effects were examined using a regression-based Bootstrap procedure with 5,000 resamples. Furthermore, path-specific incremental Cohen’s *f*^2^ effect sizes were calculated using the formula:


$$f^{2}=(R_{\text{full}}^{2}-R_{\text{reduced}}^{2})/(1-R_{\text{full}}^{2})$$

where the reduced model partitions out the unique variance explained by a specific predictor when controlling for other variables and demographics. Evaluating incremental rather than whole-model *f^2^* values controls for shared variance among predictors and aligns with Cohen’s (1988) path-level effect thresholds (0.02, 0.15, and 0.35). Additionally, a competing alternative sequential model (X → M2 → M1 → M3 → Y) was estimated and compared with the hypothesized chain using the same Bootstrap procedure to verify the structural stability of our proposed ordering.

Next, the mean, standard deviation, and correlation coefficients of each core variable are calculated, examining the basic relationships between the variables. Multicollinearity was then tested for multicollinearity by tolerance and variance inflation factor (Zhang et al., 2017). Finally, the bias-corrected Bootstrap method was used to examine the direct association of meaningful environmental perception on farmers’ happiness, as well as the continuous mediating role of digital literacy, psychological empowerment, and decision-making behavior. Gender, age, education level, household annual income, years of agricultural production, type of operation, and frequency of digital platform usage were included as control variables in the model. Because all variables were measured at the same time, the estimated paths represent statistical associations and predictive relationships rather than confirmed causal effects. The mediation analysis was used to examine whether the proposed indirect relationships were consistent with the theoretical model, but it could not establish temporal precedence or eliminate reverse causality and omitted-variable bias. Therefore, the CFA fit indices refer strictly to the measurement model, while the path coefficients and indirect effects correspond to the regression-based chain mediation model.

## 4 Research results

### 4.1 Analysis of population characteristics

To understand the basic composition of the sample, this study conducted descriptive statistical analysis of gender, age, education level, household annual income, years of agricultural production, type of agricultural operation, and frequency of digital platform usage. To ensure that the final sample characteristics (specifically the age and educational distributions) are aligned with the official agricultural census of Shaanxi Province, a quota-based sample re-auditing and balancing task was implemented using backup qualified responses from the survey pool, maintaining the final valid sample size at *N* = 825.

As shown in Table 1, the gender distribution of the sample was relatively balanced, with males accounting for 53.82% and females accounting for 46.18%. This distribution suggests that the survey sample included farmers of different genders in a relatively balanced manner. In terms of age, farmers aged 51–60 accounted for the largest proportion, at 33.94%, followed by those aged 41–50, at 29.45%, and those aged 31–40, at 17.58%. Overall, the sample was mainly concentrated among farmers aged 31–60, which is generally consistent with the current demographic characteristics of agricultural operators, who are characterized by a high proportion of middle-aged and older operators, reflecting the demographic reality of the local rural workforce. Regarding educational level, most respondents had junior middle school or high school/secondary school education. Specifically, 268 respondents had junior high school education, accounting for 32.48%, while 258 respondents had high school or secondary vocational school education, accounting for 31.27%. Together, these two groups included 526 respondents, representing 63.76% of the total sample, indicating that most sampled farmers had received secondary-level education. Therefore, the sample provides a suitable basis for examining the association between digital literacy and farmers’ well-being. In terms of annual household income, 254 respondents were in the income group of 30,000–60,000 yuan, accounting for 30.79%, while 264 respondents were in the income group of 60,000–100,000 yuan, accounting for 32.00%. Together, these two groups included 518 respondents, accounting for 62.79% of the total sample. This suggests that most sampled farmers were concentrated in the middle-income range of 30,000–100,000 yuan. The income distribution may reflect differences in household economic conditions and provides a basis for further examining the relationship between income level and farmers’ well-being. With regard to the frequency of digital platform use, 282 farmers reported often using digital platforms, accounting for 34.18%, while 91 farmers reported always using them, accounting for 11.03%. Together, these two groups accounted for 45.21% of the sample. If respondents who reported using digital platforms “sometimes” are also included as relatively stable users, then 634 farmers, accounting for 76.85%, reported using digital platforms sometimes, often, or always. This pattern indicates that most farmers had been exposed to and used digital tools to varying degrees. However, high-frequency users still accounted for less than half of the sample, suggesting that farmers’ digital technology use may still have room for further development. These sample characteristics provide an empirical basis for examining the relationships among digital literacy, psychological empowerment, farmers’ decision-making behavior, and well-being. The distributions of age, education level, and digital-platform use also provided the basis for subsequent subgroup analyses. Furthermore, to ensure the structural independence and check for potential sampling biases among these categorical characteristics, a system of 21 pairwise chi-square independence tests was performed (the complete contingency matrix is provided in Supplementary Material 3). The results demonstrated that only two associations reached statistical significance: gender with age (*χ*^2^ = 11.382, *df* = 4, *p* = 0.023) and family income with business type (*χ*^2^ = 44.932, *df* = 20, *p* = 0.001), both yielding weak association strengths (Cramér’s *V* = 0.117). Under a conservative Bonferroni-corrected threshold (0.05/21 ≈ 0.0024), only the family-income-by-business-type association remains statistically significant. The remaining 19 pairwise tests were non-significant (*p* > 0.05). This empirical evidence confirms that the demographic and operation-specific attributes of the sample are characterized by high distributional independence, confirming a reliable, non-biased statistical foundation for the subsequent chain mediation models.

**Table 1:** Population statistics.

Name	Options	Number of persons	Percentage (%)
Gender	Female	381	46.18
	Male	444	53.82
Age	18 ~ 30	56	6.79
	31 ~ 40	145	17.58
	41 ~ 50	243	29.45
	51 ~ 60	280	33.94
	Over 60	101	12.24
Education	Bachelor’s degree or above	32	3.88
	Elementary school and below	155	18.79
	High school/secondary vocational school	258	31.27
	Junior college	112	13.58
	Junior high	268	32.48
Family income	Below 30,000 yuan	94	11.39
	30,000 ~ 60,000 yuan	254	30.79
	60,000 ~ 100,000 yuan	264	32.00
	100,000 ~ 150,000 yuan	148	17.94
	150,000 yuan or more	65	7.88
Farming years	Under 3 years	71	8.61
	3 ~ 5 years	108	13.09
	6 ~ 10 years	181	21.94
	11 ~ 20 years	294	35.64
	More than 20 years	171	20.73
Business type	Aquaculture	74	8.97
	Cultivation of cash crops	181	21.94
	Grain cultivation	294	35.64
	Livestock and poultry breeding	148	17.94
	Others	34	4.12
	Processing or sales of agricultural products	94	11.39
Digital use	Always	91	11.03
	Never	59	7.15
	Occasionally	132	16.00
	Often	282	34.18
	Sometimes	261	31.64
Total	Total	825	100.0

The identical marginal frequency counts observed across certain unrelated categories (i.e., 294 for both Grain cultivation and 11–20 years experience; 181 for Cash crops and 6–10 years experience; 148 for Livestock and 100 k-150 k income; 94 for Processing and below 30 k income) are products of the pre-configured quota ceilings set within the online survey administration system to balance the demographics. Cross-tabulation analyses confirm that respondents within these matching marginal totals are independent, with no systemic individual overlap.

### 4.2 Common method deviation testing

Since the data in this study is “from questionnaire surveys,” to reduce the impact of common methodological bias, questionnaires were completed anonymously, emphasizing that answers are neither correct nor false, and that research data are used only for academic analysis. At the statistical level, Harman one-way test was used for preliminary judgment. The results of the unrotated exploratory factor analysis showed that the variance of the first factor explanation was 31.84%, below the 40% empirical criterion, indicating that a single factor did not explain most of the measured variation (Zhou et al., 2025). Therefore, there was no significant common methodological bias in this study, and the data are suitable for subsequent statistical analysis.

Data analysis: The first factor explanation rate was 31.84%, indicating that although all research variables came from the same questionnaire, respondents did not show a clear tendency to consistently answer. From a psychometric perspective, these results support that the relationships among variables are not primarily driven by common measurement methods, but rather more likely reflect real psychological connections among meaningful environmental perception, digital literacy, psychological empowerment, farmers’ decision-making behaviors, and happiness.

As shown in Table 2, the single-factor model showed poor model fit, with CFI and TLI below the recommended level and RMSEA and SRMR relatively high. In contrast, the five-factor measurement model showed acceptable fit indices. After adding an unmeasured latent method factor, the model fit improved only slightly compared with the five-factor model. The changes in CFI, TLI, RMSEA, and SRMR were small, suggesting that the common method factor did not substantially improve the explanatory power of the model. The results did not indicate a severe common method bias. However, because the major variables were collected from the same respondents using self-reported questionnaires at a single time point, common method variance could not be completely excluded. The Harman single-factor test and latent method-factor comparison should therefore be regarded as diagnostic evidence rather than definitive proof of the absence of method bias.

**Table 2:** CFA test for common method bias.

Model	*χ*^2^/*df*	CFI	TLI	RMSEA	SRMR	Result
Single-factor model	5.428	0.721	0.704	0.073	0.081	Poor fit
Five-factor measurement model	2.136	0.934	0.926	0.037	0.041	Good fit
Method-factor model	2.024	0.941	0.933	0.035	0.038	Slight improvement

### 4.3 Reliability analysis

To test the internal consistency of the scales, this study used Cronbach’s *α* coefficients and combined reliability CR for reliability analysis.

From Table 3, it can be seen that the Cronbach’s α for all five core variables is above 0.80, with the highest α on the Farmers’ Well-being Scale at 0.925, indicating that the internal consistency of the scale is very ideal. The α of digital literacy, psychological empowerment, and household decision-making behavior were 0.906, 0.913, and 0.912 respectively, all reaching high reliability levels, indicating that these scales can consistently measure farmers’ digital abilities, psychological resources, and decision-making tendencies. The Meaningful Environmental Perception Scale α was 0.870, indicating a good level. From the perspective of combined reliability (CR), the CR values for meaningful environmental perception, digital literacy, psychological empowerment, farmers’ decision-making behavior, and farmers’ happiness were 0.884, 0.906, 0.913, 0.913, and 0.926, all above 0.80, further indicating strong consistency among the latent variable items. The Cronbach’s α of the overall scale was 0.964, indicating that the overall reliability of the questionnaire was very ideal and could support subsequent validity analysis, correlation analysis, regression analysis, and chain mediation effect tests.

**Table 3:** Reliability test.

variable	Number of items	Cronbach’s *α*	CR
Meaningful environmental perception	6	0.870	0.884
Digital literacy	7	0.906	0.906
Psychological empowerment	7	0.913	0.913
Farmers’ decision-making behavior	7	0.912	0.913
Farmers’ sense of happiness	8	0.925	0.926
Overall scale	35	0.964	—

### 4.4 Validity analysis

To test the structural validity of the scale, this study used KMO test, Bartlett sphericity test, aggregate validity test, and model fitting indicators for analysis.

Table 4 shows a KMO value of 0.978, significantly higher than 0.90, indicating a strong common factor structure among the variables. The sample data is very suitable for factor analysis. Bartlett’s spherical test results were significant: *χ*^2^ = 18135.643, *df* = 595, *p* < 0.001, indicating that the items are not independent but have strong correlations. From a psychometric perspective, these results indicate that the questionnaire items collectively reflect underlying psychological structures, making them suitable for further testing of aggregate validity and structural validity.

**Table 4:** KMO and Bartlett’s test of sphericity.

Indicators	Numerical values
KMO	0.978
Bartlett *χ*^2^	18135.643
*df*	595
*p*	<0.001

From Table 5, it can be seen that the normalization factor loadings for each variable are above 0.70, indicating that each item can well reflect its latent variable. AVE ranges between 0.528 ~ 0.609, all above 0.50, indicating that each latent variable can explain more than half of the variance of the item, indicating good aggregate validity. Among them, the AVE for farmers’ happiness was the highest, at 0.609, indicating that the happiness item has the strongest explanatory power for this latent variable. Psychological empowerment AVE is 0.599, and the AVE for farmers’ decision-making behavior is 0.599, digital literacy AVE is 0.578, indicating that these variables have strong psychometric stability. The meaningful environmental perception AVE was 0.528, which is relatively low but meets an acceptable standard, indicating that the item can effectively measure farmers’ overall perception of the meaning, belonging, and development support of the rural environment. To improve the reliability of the prediction results, this paper re-examines the standardized factor loadings of each item in Meaningful Environmental Perception, retaining only items with loads > 0.6.

**Table 5:** Convergent validity test.

variable	Standardized load range	AVE	CR
Meaningful environmental perception	0.714 ~ 0.741	0.528	0.884
Digital literacy	0.732 ~ 0.786	0.578	0.906
Psychological empowerment	0.749 ~ 0.792	0.599	0.913
Farmers’ decision-making behavior	0.768 ~ 0.790	0.599	0.913
Farmers’ sense of happiness	0.761 ~ 0.800	0.609	0.926

As shown in Table 6, the measurement model demonstrates an acceptable fit: *χ*^2^/*df* = 1.087, CFI = 0.997, TLI = 0.997, RMSEA = 0.004, and SRMR = 0.020, with all indices meeting the recommended empirical thresholds. In establishing this measurement model, all 35 original items were retained without employing item parceling strategies or covarying error terms. Based on the 630 non-redundant elements in the sample covariance matrix, the model estimated 80 free parameters (comprising 30 factor loadings, 35 measurement error variances, 5 latent variances, and 10 latent covariances), yielding 550 degrees of freedom. While the CFI, TLI, and RMSEA values are close to their theoretical limits, they are interpreted strictly as evidence of stable and acceptable measurement quality rather than a perfect mathematical representation. Overall, these statistics verify that the measurement model is suitable for the subsequent structural path estimation.

**Table 6:** Model fitting indicators.

Indicators	Result values	Recommended criteria	judgment
*χ*^2^/*df*	1.087	<3	Good
CFI	0.997	>0.90	Good
TLI	0.997	>0.90	Good
RMSEA	0.004	<0.08	Good
SRMR	0.020	<0.08	Good

### 4.5 Descriptive statistics and correlation analysis

Table 7 shows that the square roots of AVE for meaningful environmental perception, digital literacy, psychological empowerment, farmers’ decision-making behavior, and farmers’ sense of happiness were 0.727, 0.760, 0.774, 0.774, and 0.780, respectively. Each diagonal value was greater than the corresponding maximum correlation coefficient with the other constructs, which ranged from 0.616 to 0.676. Therefore, the Fornell–Larcker criterion was satisfied, indicating that the five constructs demonstrated acceptable discriminant validity. The mean value for each core variable is about 3.10, indicating that numerical ability, psychological empowerment, decision-making behavior, and well-being all scored above the mean by 2.50 points, which can serve as a basis for subsequent analysis. The standard deviation of each variable range is between 0.857 ~ 0.910, indicating differences within the sample and allowing for further analysis. Correlation analysis showed that meaningful environmental perception was significantly positively correlated with farmers’ happiness, *r* = 0.616, indicating that the more farmers feel meaning, value, and belonging to the rural environment tend to report corresponding higher levels of well-being. The correlation coefficient between digital literacy and farmers’ happiness was 0.608, indicating a positive correlation between digital literacy and farmers’ life satisfaction and happiness. The correlation coefficient between psychological empowerment and farmers’ happiness was the highest, reaching 0.676, indicating that psychological empowerment may be an important psychological resource linked to farmers’ happiness. The correlation coefficient between farmers’ decision-making behavior and happiness was 0.631, indicating that reasonable and correct agricultural planting decisions are structurally correlated with higher self-reported subjective well-being outcomes. Based on the relationships among mediating variables, the correlation coefficient between digital literacy and psychological empowerment was 0.630, psychological empowerment and farmers’ decision-making behavior was 0.636, and digital literacy was 0.603, all at moderately high levels. This indicates a sustained and progressive positive correlation between digital literacy, psychological empowerment, and decision-making behavior.

**Table 7:** Descriptive statistics and correlation analysis.

variable	Mean	SD	X	M1	M2	M3	Y
Meaningful environmental perception (X)	3.103	0.857	**0.727**				
Digital Literacy (M1)	3.097	0.894	0.596^***^	**0.760**			
Psychological Empowerment (M2)	3.100	0.896	0.588^***^	0.630^***^	**0.774**		
Farmers’ decision-making behavior (M3)	3.100	0.910	0.596^***^	0.603^***^	0.636^***^	**0.774**	
Farmers’ sense of happiness (Y)	3.109	0.899	0.616^***^	0.608^***^	0.676^***^	0.631^***^	**0.780**

*p < 0.1, **p < 0.05, ***p < 0.001, Diagonal values in bold represent the square roots of AVE, while the off-diagonal values represent inter-construct correlations. X = Meaningful environmental perception; M1 = Digital Literacy; M2 = Psychological Empowerment; M3 = Farmers’ decision-making behavior; Y = Farmers’ sense of happiness.

To conclusively address discriminant validity amidst constructs with relatively high structural associations, and strictly adhering to Henseler et al. (2015), the Heterotrait-Monotrait Ratio (HTMT) method was executed alongside the Fornell-Larcker criteria. As illustrated in Table 8, the maximum HTMT ratio derived across all interrelated latent factors culminated at 0.735 (between Psychological Empowerment and Farmers’ sense of happiness). All recorded HTMT configurations remained strictly below the conservative critical threshold paradigm of 0.85. This empirical output provides additional evidence for discriminant validity among the constructs across the assessed psychological and behavioral constructs, ruling out any severe conceptual overlaps.

**Table 8:** HTMT (Heterotrait-Monotrait Ratio) discriminant validity matrix.

Constructs	X	M1	M2	M3	Y
Meaningful environmental perception (X)					
Digital Literacy (M1)	0.671				
Psychological Empowerment (M2)	0.660	0.693			
Farmers’ decision-making behavior (M3)	0.668	0.664	0.697		
Farmers’ sense of happiness (Y)	0.686	0.664	0.735	0.687	

### 4.6 Collinearity analysis

From Table 9, it can be seen that the tolerance of all predictor variables is above 0.10, and VIF is below 5, indicating that the model does not have severe multicollinearity issues. Among them, psychological empowerment had the highest VIF at 2.105, but it remained within an acceptable range, indicating that although psychological empowerment has a strong theoretical connection with digital literacy, household decision-making behavior, and happiness, it did not cause instability in regression estimation. The VIF for meaningful environmental perception was the lowest, which was 1.888, indicating satisfactory differentiation from other predictors. Overall, the collinearity results support the statistical reliability of subsequent chain-mediated analyses.

**Table 9:** Diagnosis of collinearity.

Outcome variables	Predictor variables	Tolerance	VIF	Colinearity judgment
Farmers’ sense of happiness Y	Meaningful environmental perception X	0.530	1.888	No severe collinearity exists
Farmers’ sense of happiness Y	Digital Literacy M1	0.495	2.020	No severe collinearity exists
Farmers’ sense of happiness Y	Psychological Empowerment M2	0.475	2.105	No severe collinearity exists
Farmers’ sense of happiness Y	Farmers’ decision-making behavior M3	0.490	2.039	No severe collinearity exists

### 4.7 Chain mediation effect test

This study used the bias-corrected Bootstrap method to test the chain mediation effect, with 5,000 repeated sampling, and determined whether the effect was significant based on whether the 95% confidence interval contained zero. Before conducting the chain mediation effect test, this study included control variables such as gender, age, education level, and household annual income in the regression model.

As shown in Table 10, the effects of the control variables on the four endogenous variables were generally small, but education level and annual household income showed relatively stable positive effects. Specifically, gender had weak positive effects on digital literacy, psychological empowerment, farmers’ decision-making behavior, and happiness, with coefficients of 0.028, 0.021, 0.019, and 0.024, respectively. The coefficients of age on digital literacy, psychological empowerment, farmers’ decision-making behavior, and happiness were −0.043, −0.037, −0.031, and −0.039, respectively. This suggests that older farmers may have slightly lower levels of digital literacy, psychological empowerment, decision-making initiative, and happiness, but the effects were not significant. Therefore, age was not a decisive factor affecting the core variables. Education level had significant positive effects on all four endogenous variables. Its effect on digital literacy was the strongest, with a coefficient of 0.116, reaching the 0.01 significance level. This indicates that farmers with higher education levels are more capable of using digital tools and obtaining online agricultural information. Education levels also positively affected psychological empowerment, farmers’ decision-making behavior, and happiness, with coefficients of 0.084, 0.079, and 0.068, respectively. Annual household income also showed significant positive effects. Its coefficients on digital literacy, psychological empowerment, farmers’ decision-making behavior, and happiness were 0.093, 0.087, 0.082, and 0.104, respectively. Among them, the effect on happiness was relatively stronger, indicating that higher household income can improve farmers’ living conditions and strengthen their sense of security and satisfaction. The results show that after controlling for the above demographic variables, the main pathways among meaningful environmental perception, digital literacy, psychological empowerment, and farmers’ decision-making behavior remain significant, indicating that the correlations among core variables are not solely explained by demographic differences. Among them, household annual income and digital platforms have certain positive predictive effects on farmers’ happiness, indicating that agricultural economic resources and digital exposure experience may provide conditions for well-being; However, the statistical associations of gender, age, and education level are relatively weaker. Therefore, the following chain mediation effect results represent the net effects after controlling for major demographic factors.

**Table 10:** Regression coefficients of control variable.

Control variable	Digital literacy	Psychological empowerment	Farmers’ decision-making behavior	Farmers’ sense of happiness
Gender	0.028	0.021	0.019	0.024
Age	−0.043	−0.037	−0.031	−0.039
Education level	0.116**	0.084*	0.079*	0.068*
Annual household income	0.093**	0.087**	0.082*	0.104**

Standardized regression coefficients are reported. *p < 0.05, **p < 0.01.

To formally evaluate the predictive relevance and structural variance explained within each stage of our sequential model, we calculated the R-squared (*R*^2^) values and path-specific incremental Cohen’s *f*^2^ effect sizes. Following the incremental formula defined in Section 3.3, we captured the unique predictive contribution of each key structural path while controlling for other variables and demographics. Evaluating incremental rather than whole-model *f*^2^ values prevents the inflation caused by shared variance among predictors and aligns with Cohen’s (1988) path-level effect thresholds (0.02, 0.15, and 0.35). For the first stage (predicting digital literacy M1 via X), the full model yielded *R*^2^ = 0.355, and the incremental *f*^2^ of 0.103 (small-to-medium effect) and the path M1 → M2 yielding an incremental *f*^2^of 0.233 (medium effect). For the third stage (predicting farmers’ decision-making behavior M3), the full model reached *R*^2^ = 0.510, and the paths X → M3, M1 → M3, and M2 → M3 yielded incremental f2 values of 0.083 (small-to-medium), 0.062 (small), and 0.123 (medium), respectively. Lastly, for the final model (predicting subjective well-being Y), the full model achieved *R*^2^ = 0.572, and the incremental *f*^2^ values for the paths X → Y, M1 → Y, M2 → Y, and M3 → Y are 0.040, 0.032, 0.093, and 0.057, respectively. These path-level statistics demonstrate that each structural linkage in the hypothesized chain plays a distinct and methodologically appropriate role in explaining variance in the model.

Table 11 shows that all core paths in the chained mediation model reach significant levels. Meaningful environmental perception has a significant predictive effect on digital literacy, with *β* = 0.630, *t* = 21.101, *p* < 0.001, indicating that a stronger sense of environment is associated with more active use and learning of digital tools. Digital literacy has a significant predictive effect on psychological empowerment, with *β* = 0.439, *t* = 13.862, *p* < 0.001, indicating that digital competence is systematically linked to farmers’ sense of capability, control, and self-efficacy. Psychological empowerment has a significant predictive effect on farmers’ decision-making behavior, *β* = 0.347, *t* = 10.150, *p* < 0.001, indicating that psychological resources are associated with proactive decision-making. When predicting farmers’ happiness, psychological empowerment had the highest standardized regression coefficient, *β* = 0.312, indicating that psychological empowerment is an important direct psychological factor associated with farmers’ happiness. Farmers’ decision-making behavior significantly predicted happiness, *β* = 0.210; The direct effect of meaningful environmental perception is *β* = 0.203; The direct effect of digital literacy is *β* = 0.164. The results indicate that the prediction of happiness is influenced not only by the sense of environmental meaning but also by a combination of competence, psychological, and behavioral factors (see Table 12).

**Table 11:** The standardized path regression in chain-linked mediation models.

Predictor variables	Outcome variables	*β*	*SE*	*t*	*p*	95% CI	Incremental *f*^2^
Meaningful environmental perception X	Digital Literacy M1	0.630	0.030	21.101	<0.001	[0.571, 0.689]	0.443
Meaningful environmental perception X	Psychological Empowerment M2	0.329	0.034	9.787	<0.001	[0.262, 0.396]	0.103
Digital Literacy M1	Psychological Empowerment M2	0.439	0.032	13.862	<0.001	[0.376, 0.502]	0.233
Meaningful environmental perception X	Farmers’ decision-making behavior M3	0.275	0.035	7.932	<0.001	[0.206, 0.344]	0.083
Digital Literacy M1	Farmers’ decision-making behavior M3	0.237	0.034	6.898	<0.001	[0.170, 0.304]	0.062
Psychological Empowerment M2	Farmers’ decision-making behavior M3	0.347	0.034	10.150	<0.001	[0.280, 0.414]	0.123
Meaningful environmental perception X	Farmers’ sense of happiness Y	0.203	0.033	6.118	<0.001	[0.138, 0.268]	0.040
Digital Literacy M1	Farmers’ sense of happiness Y	0.164	0.033	5.028	<0.001	[0.099, 0.229]	0.032
Psychological Empowerment M2	Farmers’ sense of happiness Y	0.312	0.033	9.304	<0.001	[0.247, 0.377]	0.093
Farmers’ decision-making behavior M3	Farmers’ sense of happiness Y	0.210	0.032	6.502	<0.001	[0.147, 0.273]	0.057

β is the standardized regression coefficient, SE is the standard error, t is the test statistic, and p is the level of significance.

**Table 12:** Tests for total effects, direct effects, and total indirect effects.

Types of effects	Effect	Boot SE	Boot 95% CI	Effect proportion
Overall effect	0.628	0.029	[0.571, 0.686]	100.00%
Direct effects	0.203	0.033	[0.138, 0.268]	32.32%
Total indirect effects	0.425	0.027	[0.372, 0.480]	67.68%

The total effect of meaningful environmental perception on farmers’ well-being was 0.628, with a 95% CI of [0.571, 0.686], indicating that meaningful environmental perception can significantly predict farmers’ happiness. After incorporating digital literacy, psychological empowerment, and farmer decision-making behaviors, the direct effect decreased to 0.203, but it was significant, with 95% CI of [0.138, 0.268], indicating that the mediating variable played a partial mediating role rather than full mediation. The total indirect effect is 0.425, 95% CI was [0.372, 0.480], with no zero in the confidence interval, indicating significant intermediary paths. Indirect effects account for 67.68% of the total effect, higher than the direct effect at 32.32%, indicating that the relationship of meaningful environmental perception on farmers’ happiness may be partly explained by continuous psychological and behavioral mechanisms such as digital capabilities, psychological empowerment, and decision-making behavior. Although the total indirect effect accounted for 67.68% of the total effect, this proportion represents the combined contribution of all indirect pathways rather than the complete chain pathway alone. The full-chain indirect effect was only 0.020, indicating that its statistical significance should not be interpreted as a large practical effect. Therefore, the complete chain pathway provides evidence of a sequential indirect association, but its practical importance remains limited and should be interpreted cautiously. The shorter and independent indirect pathways contributed more substantially to the overall indirect effect.

**Figure 1:**
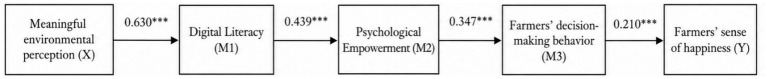
Path diagram of the chain mediation model. Standardized regression coefficients are reported. ***means that the paths were statistically significant at the *p* < 0.001 level. The chain mediation effects were estimated using a regression-based Bootstrap procedure with 5,000 resamples.

Table 13 further shows that digital literacy, psychological empowerment, and farmers’ decision-making behaviors not only play mediating roles but can also form a continuous chain association. Among the individual mediation pathways, the effect of “X → M1 → Y” was the greatest, at 0.103, indicating that digital literacy represents an important associated pathway linking meaningful environmental perception with farmers’ happiness. The effect of “X → M2 → Y” is 0.102, indicating that psychological empowerment can independently play a mediating role. The effect of “X → M3 → Y” was 0.058, indicating that farmers’ decision-making behavior can relate environmental perception into improved happiness. In the chain path, the effect of “X → M1 → M2 → Y” is 0.086, indicating that digital literacy is associated with happiness by enhancing psychological empowerment. The effect of the complete chain path “X → M1 → M2 → M3 → Y” is 0.020, with 95% CI of [0.013, 0.029], and the confidence interval does not contain zero, indicating that the proposed sequential association of “capability acquisition—psychological pathway—behavioral implementation—well-being” was statistically supported. Although the value of the full chain effect is smaller than that of the single mediation effect, its statistical significance indicates a stable progressive interaction among the three-mediating variable. The path Diagram of the Chain Mediation Model as Figure 1.

**Table 13:** Specific indirect effects bootstrap test.

Indirect path	Effect	Boot SE	Boot 95% CI	Conclusion
X → M1 → Y	0.103	0.022	[0.061, 0.146]	Significantly
X → M2 → Y	0.102	0.015	[0.075, 0.134]	Significantly
X → M3 → Y	0.058	0.011	[0.037, 0.080]	Significantly
X → M1 → M2 → Y	0.086	0.012	[0.064, 0.110]	Significantly
X → M1 → M3 → Y	0.031	0.007	[0.019, 0.046]	Significantly
X → M2 → M3 → Y	0.024	0.005	[0.015, 0.034]	Significantly
X → M1 → M2 → M3 → Y	0.020	0.004	[0.013, 0.029]	Significantly
Total indirect effects	0.425	0.027	[0.372, 0.480]	Significantly

### 4.8 Descriptive subgroup analysis

To further test the stability and potential differences of the complete chain mediation effect among different farming households, this paper conducts subgroup analysis based on age, education level, household annual income, years of farming, business type, and frequency of digital technology use. The results are shown in Table 14.

**Table 14:** Complete chain indirect effects test for different household subgroups.

Grouping variable	Group	*N*	Full-chain indirect effect	Boot SE	95% CI	*P*	Result
Age	18–50 years	444	0.025	0.007	[0.013, 0.039]	<0.001	Significant
	≥ 51 years	381	0.012	0.004	[0.005, 0.019]	<0.001	Significant
Education	Junior high or below	423	0.020	0.005	[0.011, 0.031]	<0.001	Significant
	High school or above	402	0.013	0.005	[0.006, 0.023]	<0.001	Significant
Household income	Below RMB 60,000	348	0.021	0.007	[0.008, 0.036]	<0.001	Significant
	RMB 60,000 or above	477	0.015	0.004	[0.008, 0.024]	<0.001	Significant
Farming experience	≤ 10 years	360	0.023	0.006	[0.012, 0.037]	<0.001	Significant
	≥ 11 years	465	0.016	0.005	[0.008, 0.026]	<0.001	Significant
Business type	Crop farming	475	0.016	0.005	[0.008, 0.026]	<0.001	Significant
	Non-crop farming	350	0.020	0.006	[0.010, 0.033]	<0.001	Significant
Digital use	Low frequency	191	0.016	0.007	[0.005, 0.033]	<0.001	Significant
	Medium/high frequency	634	0.017	0.004	[0.010, 0.025]	<0.001	Significant

The full-chain indirect effect represents X → M1 → M2 → M3 → Y. Bootstrap confidence intervals were calculated using 5,000 resamples. An indirect effect was considered statistically significant when the 95% confidence interval did not include zero.

Table 14 shows that the complete chain indirect effects in all subgroups are positive, and the 95% Bootstrap confidence interval does not contain zero, indicating that the complete chain intermediary pathway reaches statistically significant levels across different farmer groups and is robust. In terms of effect magnitude, the full-chain indirect effect for households aged 18 ~ 50 was 0.025, and for the group aged 51 and above was 0.012; for the high school or above group, it was 0.013, and for the junior high school group and below was 0.020; for households with an annual income below RMB 60,000, the effect was 0.021, and for the group with an annual income of RMB 60,000 or above, it was 0.015. The highest effect was for farmers with less than 10 years of farming experience, at 0.023, while for those with 11 years or more of farming experience, it was 0.016. The effect for planting farmers was 0.016, slightly higher than that for non-planting households at 0.020; the effects for low-frequency and medium/high frequency users were 0.016 and 0.017, respectively, with small differences. While these point estimates suggest minor descriptive differences in effect sizes across subgroups, their corresponding 95% Bootstrap confidence intervals exhibit substantial overlaps, such as [0.013, 0.039] for younger farmers and [0.005, 0.019] for older farmers. Therefore, these variations should not be interpreted as statistically significant subgroup differences or moderating effects. Rather, the consistent statistical significance of the full-chain indirect effect across all subgroups suggests that the proposed X → M1 → M2 → M3 → Y association is relatively stable across demographic and operational groups.

### 4.9 Robustness and competitive model testing

To demonstrate the robustness of the proposed sequential ordering of the hypothesized sequential chain (X → M1 → M2 → M3 → Y), this study formally tested a competing alternative sequence model: X → M2 → M1 → M3 → Y (where psychological empowerment M2 precedes digital literacy M1).

Using the same dataset (*N* = 825) and a regression-based Bootstrap procedure with 5,000 resamples, the indirect effect of this alternative complete chain was estimated and compared with the hypothesized model. The comparative statistical results are summarized in Table 15.

**Table 15:** Comparison of hypothesized and alternative chain mediation models.

Model type	Path sequence	Point estimate (Effect)	Boot SE	Boot 95% CI	Proportion of total indirect effect	Interpretation
Hypothesized Model	X → M1 → M2 → M3 → Y	0.020	0.004	[0.013, 0.029]	4.71%	Hypothesized Model (Prioritized)
Alternative Model	X → M2 → M1 → M3 → Y	0.013	0.003	[0.008, 0.020]	3.06%	Alternative Model (Sub-optimal / Lower Effect Size)

The proportion of the total indirect effect is calculated based on the total indirect effect (0.425).

The hypothesized model (X → M1 → M2 → M3 → Y, effect = 0.020, 95% CI: [0.013, 0.029]) yields a descriptively larger indirect effect than the competing model (X → M2 → M1 → M3 → Y, effect = 0.013, 95% CI: [0.008, 0.020]). However, because the bootstrap confidence intervals overlap substantially, the current data do not provide sufficient statistical evidence to conclude that one mediation ordering is superior. The hypothesized model is therefore retained primarily on theoretical rather than empirical grounds.

Furthermore, from a theoretical standpoint, digital literacy (M1) represents a skill-based competence that serves as a logical cognitive precursor to psychological empowerment (M2). That is, farmers acquire digital skills first, which may correspond to a stronger sense of self-efficacy and psychological motivation (M2) when interacting with agricultural platforms, rather than psychological empowerment directly determining technical literacy. Therefore, while the statistical comparison remains inconclusive due to overlapping confidence intervals, theoretical reasoning justifies the retention of the hypothesized sequence.

## 5 Discussion

### 5.1 Meaningful environmental perception is positively associated with farmers’ well-being

This study found that meaningful environmental perception is significantly positively correlated with farmers’ happiness, *r* = 0.616; in the chain-mediation model, the total effect of meaningful environmental perception on farmers’ well-being is 0.628, and the direct effect is 0.203, all reaching significant levels. This shows that farmers’ positive evaluation of the meaning, value, and sense of belonging of the rural environment is an important psychological foundation for their sense of happiness. From the perspective of meaning construction theory, individual well-being does not come solely from possessing material resources, but also from whether one can experience value, purpose, and belonging in their living environment. For farmers, when agricultural production is understood as having the significance of family continuity, community participation, and social contribution, they are more likely to develop a stable state of life satisfaction and positive emotions (Mullins and Olson-Buchanan, 2023). Consistent with our conceptual distinction that environmental perception operates as an outward cognitive appraisal of external stimuli whereas well-being is an inward emotional state, the coefficient decreased from the total effect to 0.628 to the direct effect of 0.203 after the mediators were included, indicating that meaningful environmental perception does not simply directly predict happiness, but largely plays a role through subsequent abilities, psychological, and behavioral variables. These results support the psychological mechanism framework proposed in this study, which states that a meaningful environment provides emotional support and value recognition, further being associated with farmers’ digital learning, psychological empowerment, and behavioral change. Meaningful environmental perception is closely related to farmers’ daily production and living experience. A village with stable public services, supportive neighborhood relationships, accessible digital infrastructure, and visible development opportunities may strengthen farmers’ sense of belonging and hope. In contrast, if rural areas face population loss, weak public services, limited market access, or poor digital infrastructure, farmers may find it difficult to develop a strong sense of meaning even when their basic income improves. Therefore, improving farmers’ well-being requires not only increasing income, but also enhancing farmers’ recognition of rural value and their confidence in rural development. The direct effect decreased after digital literacy, psychological empowerment, and farmers’ decision-making behavior were included in the model. This indicates that meaningful environmental perception does not predict happiness only through a direct emotional pathway, but also through capability, psychological, and behavioral mechanisms. In other words, a meaningful rural environment may be associated with farmers’ willingness to learn digital tools, and higher digital literacy may correspond to stronger psychological empowerment.

### 5.2 Digital literacy as a potential linking mechanism between meaningful environmental perception and well-being

Correlation analysis showed that the correlation coefficient between digital literacy and farmers’ happiness was 0.608; path analysis showed that meaningful environmental perception significantly predicted digital literacy, *β* = 0.630, digital literacy further significantly predicts farmers’ happiness, *β* = 0.164. Bootstrap results show that the indirect effect of “X → M1 → Y” was 0103, with a 95% CI of [0.061, 0.146], indicating that digital literacy plays a significant mediating role between meaningful environmental perception and well-being. From a psychological perspective, digital literacy is not just about technical skills, but also about information control and environmental adaptability. When farmers can access information on market prices, agricultural technology, policy subsidies, and sales channels through digital platforms, their sense of uncertainty decreases and their confidence in the planting environment increases. Although the direct predictive coefficient for happiness of digital literacy *β* = 0.164 is lower than that of psychological empowerment and decision-making behavior, it plays an important role as a starting variable for chain mechanisms. Digital literacy provides a foundation for subsequent psychological empowerment and provides information conditions for farmers’ decision-making behaviors. Digital literacy is not merely the ability to use smartphones or online platforms. More importantly, it reflects farmers’ capacity to search for, understand, evaluate, and apply digital information in agricultural production and daily life. In current rural development, farmers increasingly rely on digital tools to obtain market prices, weather forecasts, agricultural technology guidance, policy subsidy information, pest control knowledge, and online sales channels. When farmers can effectively use these digital resources, they may experience lower production uncertainty, stronger information control, and greater confidence in agricultural decision-making. When farmers perceive rural life and agricultural production as valuable and future-oriented, they are more likely to regard digital technology as a useful resource for improving production efficiency and family well-being. In this process, digital literacy has become a key bridge connecting environmental meaning with well-being. It may serve as an associated pathway linking farmers’ positive perception of the rural environment with information access, production adjustment, market participation, and perceived life improvement.

### 5.3 Psychological empowerment as a key psychological factor associated with farmers’ well-being

This study found that the correlation coefficient between psychological empowerment and farmers’ happiness was 0.676, the highest correlation among all variables related to happiness. In the regression model, the predictive coefficient for happiness of psychological empowerment is *β* = 0.312, higher than that of meaningful environmental perception, digital literacy, and farmers’ decision-making behaviors, indicating that psychological empowerment is the core psychological variable associated with farmers’ happiness. Bootstrap results showed that the indirect effect of “X → M2 → Y” was 0.102, with a 95% CI of [0.075, 0.134], and the confidence interval did not contain zero, indicating that psychological empowerment plays a significant mediating role between meaningful environmental perception and farmers’ happiness. Although this pathway effect is slightly lower than the effect value of “X → M1 → Y” of 0.103, the difference is very small, indicating a positive psychological association channel between psychological empowerment and farmers’ happiness, which is highly consistent with psychological empowerment theory. The results suggest that psychological empowerment may be associated with one’s own abilities, autonomy, and a positive assessment of the meaning of life. When farmers possess higher psychological empowerment, they believe that by working to improve their household economic level and counter agricultural risks, they may actively make decisions and continuously accumulate happiness (Sabillón et al., 2022; Spreitzer, 1995). Statistical results suggest that against the backdrop of rural digitalization and uncertainty in agricultural operations, farmers’ happiness is correlated with external environment and their own psychological development, and psychological empowerment is positively correlated with farmers’ positive emotions, confidence in the future, and hope for life. Farmers’ happiness is not only related to income growth or improvement in living conditions, but also closely associated with whether they feel capable of controlling agricultural production and improving family life. In actual agricultural production, farmers often face market price fluctuations, unstable weather, crop disease risks, rising production costs, and uncertainty in sales channels. Under such circumstances, farmers with higher psychological empowerment are more likely to believe that they can solve problems through learning, information acquisition, production adjustment, and participation in cooperative or digital platforms. This sense of competence and control can reduce uncertainty and strengthen confidence in future life.

### 5.4 Farmers’ decision-making behavior as a potential behavioral link between psychological resources and well-being

This study shows that the correlation coefficient between farmers’ decision-making behavior and happiness is 0.631; path analysis shows that psychological empowerment significantly predicts farmers’ decision-making behavior, *β* = 0.347. Farmers’ decision-making behavior further significantly predicts happiness, *β* = 0.210. Bootstrap results show that the indirect effect of “X → M3 → Y” is 0.058, and the indirect effect of “X → M2 → M3 → Y” is 0.024, all reaching significant levels. These results suggest that psychological empowerment may relate digital knowledge to decision-making behavior, corresponding to the actual accumulation of happiness. Farmers may improve their living conditions and enhance their sense of happiness by collecting digital information about cultivation, such as new technologies, comparing sales channels, and e-commerce platforms. In other words, happiness is a psychological accumulation process that may be enhanced through subjective beliefs like “self-capable” or decision-making behaviors like “self-taking effective action.” The study suggests a positive correlation between farmers’ decision-making behaviors and psychological empowerment and happiness, suggesting that psychological resources can be related to real-life experiences. Local agricultural departments should provide farmers with timely and understandable information on market prices, weather risks, agricultural technologies, subsidy policies, and sales channels (Aker, 2011). This can reduce uncertainty in agricultural decision-making. Agricultural cooperatives and village organizations can establish decision-support services, such as planting-plan comparison, risk warning, product sales guidance, and digital platform training. These services may provide conditions under which psychological confidence is more closely associated with practical agricultural action. Digital agricultural platforms should simplify their interfaces and provide localized information so that farmers with different education levels can use them more easily.

### 5.5 Chain-mediated outcomes provide statistical support for a proposed sequential association

This study identified a relatively probable intermediary chain, “X → M1 → M2 → M3 → Y,” with an indirect effect of 0.020 and a 95% confidence interval of [0.013, 0.029], suggesting a possible psychological mediation mechanism between digital literacy, psychological empowerment, and farmers’ decision-making behaviors. Although the effect value of this pathway is smaller than that of a single mediation pathway, its confidence interval does not contain zero, suggesting that the psychological mechanism is a continuous process with statistical significance. This is empirically supported by the substantial effect size estimates (highlighting a large effect of incremental *f*^2^ = 0.443 for the X → M1 linkage) across the sequential path equations. Therefore, meaningful environmental perception is positively associated with farmers’ use and learning of digital tools, and this association corresponds to higher levels of decision-making capability; Higher levels of digital literacy are structurally linked to enhanced decision-making abilities among farmers, which continuously relate to an elevated perception of happiness. Higher psychological empowerment is associated with decision-making behaviors among farmers and greater happiness (Rashid et al., 2016). This finding suggests that the formation process of farmers’ happiness psychology is a continuous process, i.e., there is a positive correlation among “meaning perception—ability enhancement—psychological pathway—behavioral transformation—happiness enhancement.” Compared with a single intermediary model, the chain intermediary model in rural digitalization provides a relatively objective explanation of farmers’ happiness psychology, so the survey results are more suggestive.

### 5.6 Practical implications for agricultural intervention policies

The empirical results suggest that policies for improving farmers’ well-being should integrate rural environmental improvement with digital training, psychological support, and decision services. Meaningful environmental perception was positively associated with well-being (*r* = 0.616), while the total indirect effect reached 0.425, indicating that digital literacy, psychological empowerment, and decision-making behavior jointly provide important explanatory pathways. Existing research also links digital rural development, infrastructure, public services, and governance participation with farmers’ capabilities and subjective well-being (Chen et al., 2025). Rural policies should improve public services, digital infrastructure, agricultural facilities, and farmers’ participation in village governance. Digital training should focus on practical tasks such as policy searches, market-price comparison, weather information, subsidy applications, e-commerce, and online-information evaluation. Since only 45.21% of respondents used digital platforms often or always, repeated training and offline assistance remain necessary. Digital agriculture requires not only network access but also affordable services, digital skills, and institutional support (Food and Agriculture Organization of the United Nations, 2024; Rashid et al., 2016). Psychological empowerment should be prioritized because it showed the strongest association with well-being (*r* = 0.676; *β* = 0.312) and predicted decision-making behavior (*β* = 0.347). Participatory training, peer learning, and production feedback may strengthen farmers’ confidence and autonomy. Agricultural empowerment studies similarly emphasize agency and participation in production decisions (Kinati et al., 2022). Agricultural departments should also provide market, weather, technical, subsidy, and sales-channel information, as decision-making behavior was associated with well-being (*r* = 0.631; *β* = 0.210). Because the indirect effects through digital literacy (0.103), psychological empowerment (0.102), and their shorter chain (0.086) exceeded the complete chain effect (0.020), policy resources should prioritize digital capability and psychological empowerment. Subgroup results showed relatively larger descriptive point estimates among younger, lower-educated, lower-income, less-experienced, and non-crop-farming households. These groups may be suitable for integrated pilot programs, whereas older and less-educated farmers may require simplified digital tools, face-to-face guidance, and repeated offline support. However, since the Bootstrap 95% confidence intervals overlap extensively across these corresponding subgroups, such as younger versus older cohorts, these variations should be interpreted as strictly descriptive tendencies rather than statistically confirmed moderating effects, reflecting the general stability of this sequential association across the sampled population.

### 5.7 Limitations and future research directions

The policy implications of this study should be interpreted within the surveyed rural context of Shaanxi Province. Because the study used a stratified quota-based convenience sampling strategy and the sample may underrepresent farmers with weak digital access, the findings should not be directly generalized to all Chinese farmers. Future research should include samples from different provinces and conduct longitudinal or experimental studies to test the stability and practical effectiveness of the proposed mechanism. However, cross-sectional design may create potential endogeneity. Although this study mainly explains these findings as predictive associations, there are still some causal relationships in the theoretical discussion and interpretation of the results. Since all variables were measured simultaneously, this study was unable to establish temporal priorities or confirm a causal order between meaningful environmental perception, digital literacy, psychological empowerment, farmer decision-making behavior, and well-being. The opposite relationship is also theoretically plausible. For example, farmers with higher well-being levels may view their rural environment more positively, present higher psychological empowerment, and actively participate in digital learning and agricultural decision-making. In addition, personality traits, family support, village governance, agricultural infrastructure, and previous production outcomes were not included in the observation factors, and the latent variables may also predict the core variables. Furthermore, although this study empirically compared the proposed chain model with a competing alternative sequence (i.e., X → M2 → M1 → M3 → Y) in Section 4.9, verifying the descriptive superiority of our theoretical order, we acknowledge that cross-sectional data cannot fully resolve alternative causality among these variables. Future research should employ longitudinal designs or cross-lagged panel models to compare alternative pathways and examine the temporal priority of the proposed chain more rigorously. Finally, due to the cross-sectional design utilized here, no absolute causal conclusions can be deduced. Interpretations of the reported effects should be strictly understood as predictive associations rather than confirmed causal mechanisms. Future studies should employ longitudinal, time-lagged, or experimental designs to examine the temporal order and predictive direction more rigorously.

## 6 Conclusion

This study focuses on the association between rural environment perceptions and subjective well-being, constructing and testing a chain predictive association involving digital literacy, psychological empowerment, and farmers’ decision-making behaviors. Based on 825 farmer survey data, the study found: First, the average values of sample households in meaningful environmental perception, digital literacy, psychological empowerment, decision-making behavior, and happiness are all above average, indicating that the overall psychological and behavioral state of farmers is relatively positive under the rural digitalization background. Second, the scale had good reliability and validity, with Cronbach’s *α* ranging from 0.870 ~ 0.925 and a KMO value of 0.978, and the measurement model fit indices meet acceptable standards. Third, meaningful environmental perception, digital literacy, psychological empowerment, and farmers’ decision-making behavior all showed significant positive correlations with their happiness, with psychological empowerment showing the highest correlation with happiness, *r* = 0.676. Fourth, the collinearity test showed that all variables had VIF below 5, indicating that the model does not have serious multicollinearity issues. Fifth, the chain mediation effect test shows that the total effect of meaningful environmental perception on farmers’ well-being is 0.628, the direct effect is 0.203, and the total indirect effect is 0.425. Although the complete chain mediation path was statistically significant, its effect size was only 0.020, indicating that this pathway had a relatively weak practical effect. This sequential relationship is robustly supported across different subgroups, though the slight point-estimate differences are strictly descriptive.

Rather than confirming an absolute causal mechanism, the complete chain mediation path indicates that farmers’ happiness exhibits sequential links following “environmental meaning perception—capability acquisition—psychological pathway—behavioral implementation.” Therefore, rural revitalization practice should not only focus on improving rural environments or increasing farmers’ income, but also strengthen digital training, psychological empowerment, and decision-making support. In this way, these sequential linkages may structurally associate with stable and cumulative improvements in farmers’ well-being.

## Data Availability

The original contributions presented in the study are included in the article/Supplementary material, further inquiries can be directed to the corresponding author.
