# Supplementary material for: Factors associated with farmers’ well-being in meaningful environments: a chain psychological association of digital literacy, psychological empowerment, and farmers’ decision-making behaviors
Source: Front Psychol. 2026 Jul 28;17:1891582. doi: 10.3389/fpsyg.2026.1891582 (PMC13457035; doi:10.3389/fpsyg.2026.1891582)
Supplement: Supplementary file 1 [file Data_Sheet_1.ZIP › Supplementary_Material_3_Crosstabs.docx]

| **Table 1. Cross-Tabulation and Chi-Square Test of Age by Gender** | | | | | | |
| --- | --- | --- | --- | --- | --- | --- |
| **Variable** | **Category** | **Gender, n (%)** | | **Total, n (%)** | **χ²** | **p** |
|  |  | Female | Male |  |  |  |
| Age | 18–30 | 16(4.20) | 40(9.01) | 56(6.79) | 11.382 | 0.023 |
|  | 31–40 | 74(19.42) | 71(15.99) | 145(17.58) |  |  |
|  | 41–50 | 110(28.87) | 133(29.95) | 243(29.45) |  |  |
|  | 51–60 | 126(33.07) | 154(34.68) | 280(33.94) |  |  |
|  | Over 60 | 55(14.44) | 46(10.36) | 101(12.24) |  |  |
| Total | | 381 | 444 | 825 |  |  |
| Note. Values are presented as n (%). * p < .10, ** p < .05, *** p < .01. | | | | | | |

| **Table 2. Cross-Tabulation and Chi-Square Test of Education level by Gender** | | | | | | |
| --- | --- | --- | --- | --- | --- | --- |
| **Variable** | **Category** | **Gender, n (%)** | | **Total, n (%)** | **χ²** | **p** |
|  |  | Female | Male |  |  |  |
| Education level | Bachelor’s degree or above | 19(4.99) | 13(2.93) | 32(3.88) | 5.283 | 0.260 |
|  | Elementary school and below | 74(19.42) | 81(18.24) | 155(18.79) |  |  |
|  | High school/secondary vocational school | 109(28.61) | 149(33.56) | 258(31.27) |  |  |
|  | Junior college | 48(12.60) | 64(14.41) | 112(13.58) |  |  |
|  | Junior high | 131(34.38) | 137(30.86) | 268(32.48) |  |  |
| Total | | 381 | 444 | 825 |  |  |
| Note. Values are presented as n (%). * p < .10, ** p < .05, *** p < .01. | | | | | | |

| **Table 3. Cross-Tabulation and Chi-Square Test of Family income by Gender** | | | | | | |
| --- | --- | --- | --- | --- | --- | --- |
| **Variable** | **Category** | **Gender, n (%)** | | **Total, n (%)** | **χ²** | **p** |
|  |  | Female | Male |  |  |  |
| Family income | 100,000–150,000 yuan | 70(18.37) | 78(17.57) | 148(17.94) | 1.095 | 0.895 |
|  | 150,000 yuan or more | 31(8.14) | 34(7.66) | 65(7.88) |  |  |
|  | 30,000 to 60,000 yuan | 120(31.50) | 134(30.18) | 254(30.79) |  |  |
|  | 60,000–100,000 yuan | 121(31.76) | 143(32.21) | 264(32.00) |  |  |
|  | Below 30,000 yuan | 39(10.24) | 55(12.39) | 94(11.39) |  |  |
| Total | | 381 | 444 | 825 |  |  |
| Note. Values are presented as n (%). * p < .10, ** p < .05, *** p < .01. | | | | | | |

| **Table 4. Cross-Tabulation and Chi-Square Test of Farming experience by Gender** | | | | | | |
| --- | --- | --- | --- | --- | --- | --- |
| **Variable** | **Category** | **Gender, n (%)** | | **Total, n (%)** | **χ²** | **p** |
|  |  | Female | Male |  |  |  |
| Farming experience | 11–20 years | 134(35.17) | 160(36.04) | 294(35.64) | 1.396 | 0.845 |
|  | 3–5 years | 49(12.86) | 59(13.29) | 108(13.09) |  |  |
|  | 6–10 years | 86(22.57) | 95(21.40) | 181(21.94) |  |  |
|  | More than 20 years | 83(21.78) | 88(19.82) | 171(20.73) |  |  |
|  | Under 3 years | 29(7.61) | 42(9.46) | 71(8.61) |  |  |
| Total | | 381 | 444 | 825 |  |  |
| Note. Values are presented as n (%). * p < .10, ** p < .05, *** p < .01. | | | | | | |

| **Table 5. Cross-Tabulation and Chi-Square Test of Business type by Gender** | | | | | | |
| --- | --- | --- | --- | --- | --- | --- |
| **Variable** | **Category** | **Gender, n (%)** | | **Total, n (%)** | **χ²** | **p** |
|  |  | Female | Male |  |  |  |
| Business type | Aquaculture | 36(9.45) | 38(8.56) | 74(8.97) | 7.233 | 0.204 |
|  | Cultivation of cash crops | 85(22.31) | 96(21.62) | 181(21.94) |  |  |
|  | Grain cultivation | 149(39.11) | 145(32.66) | 294(35.64) |  |  |
|  | Livestock and poultry breeding | 57(14.96) | 91(20.50) | 148(17.94) |  |  |
|  | Others | 13(3.41) | 21(4.73) | 34(4.12) |  |  |
|  | Processing or sales of agricultural products | 41(10.76) | 53(11.94) | 94(11.39) |  |  |
| Total | | 381 | 444 | 825 |  |  |
| Note. Values are presented as n (%). * p < .10, ** p < .05, *** p < .01. | | | | | | |

| **Table 6. Cross-Tabulation and Chi-Square Test of Digital platform use frequency by Gender** | | | | | | |
| --- | --- | --- | --- | --- | --- | --- |
| **Variable** | **Category** | **Gender, n (%)** | | **Total, n (%)** | **χ²** | **p** |
|  |  | Female | Male |  |  |  |
| Digital platform use frequency | Always | 42(11.02) | 49(11.04) | 91(11.03) | 3.174 | 0.529 |
|  | Never | 31(8.14) | 28(6.31) | 59(7.15) |  |  |
|  | Occasionally | 57(14.96) | 75(16.89) | 132(16.00) |  |  |
|  | Often | 138(36.22) | 144(32.43) | 282(34.18) |  |  |
|  | Sometimes | 113(29.66) | 148(33.33) | 261(31.64) |  |  |
| Total | | 381 | 444 | 825 |  |  |
| Note. Values are presented as n (%). * p < .10, ** p < .05, *** p < .01. | | | | | | |

| **Table 7. Cross-Tabulation and Chi-Square Test of Education level by Age** | | | | | | | | | |
| --- | --- | --- | --- | --- | --- | --- | --- | --- | --- |
| **Variable** | **Category** | **Age, n (%)** | | | | | **Total, n (%)** | **χ²** | **p** |
|  |  | 18–30 | 31–40 | 41–50 | 51–60 | Over 60 |  |  |  |
| Education level | Bachelor’s degree or above | 4(7.14) | 4(2.76) | 8(3.29) | 12(4.29) | 4(3.96) | 32(3.88) | 20.186 | 0.212 |
|  | Elementary school and below | 16(28.57) | 24(16.55) | 49(20.16) | 46(16.43) | 20(19.80) | 155(18.79) |  |  |
|  | High school/secondary vocational school | 18(32.14) | 52(35.86) | 83(34.16) | 82(29.29) | 23(22.77) | 258(31.27) |  |  |
|  | Junior college | 8(14.29) | 19(13.10) | 26(10.70) | 46(16.43) | 13(12.87) | 112(13.58) |  |  |
|  | Junior high | 10(17.86) | 46(31.72) | 77(31.69) | 94(33.57) | 41(40.59) | 268(32.48) |  |  |
| Total | | 56 | 145 | 243 | 280 | 101 | 825 |  |  |
| Note. Values are presented as n (%). * p < .10, ** p < .05, *** p < .01. | | | | | | | | | |

| **Table 8. Cross-Tabulation and Chi-Square Test of Family income by Age** | | | | | | | | | |
| --- | --- | --- | --- | --- | --- | --- | --- | --- | --- |
| **Variable** | **Category** | **Age, n (%)** | | | | | **Total, n (%)** | **χ²** | **p** |
|  |  | 18–30 | 31–40 | 41–50 | 51–60 | Over 60 |  |  |  |
| Family income | 100,000–150,000 yuan | 11(19.64) | 28(19.31) | 42(17.28) | 46(16.43) | 21(20.79) | 148(17.94) | 8.792 | 0.922 |
|  | 150,000 yuan or more | 6(10.71) | 9(6.21) | 20(8.23) | 22(7.86) | 8(7.92) | 65(7.88) |  |  |
|  | 30,000 to 60,000 yuan | 19(33.93) | 48(33.10) | 72(29.63) | 87(31.07) | 28(27.72) | 254(30.79) |  |  |
|  | 60,000–100,000 yuan | 16(28.57) | 47(32.41) | 82(33.74) | 92(32.86) | 27(26.73) | 264(32.00) |  |  |
|  | Below 30,000 yuan | 4(7.14) | 13(8.97) | 27(11.11) | 33(11.79) | 17(16.83) | 94(11.39) |  |  |
| Total | | 56 | 145 | 243 | 280 | 101 | 825 |  |  |
| Note. Values are presented as n (%). * p < .10, ** p < .05, *** p < .01. | | | | | | | | | |

| **Table 9. Cross-Tabulation and Chi-Square Test of Farming experience by Age** | | | | | | | | | |
| --- | --- | --- | --- | --- | --- | --- | --- | --- | --- |
| **Variable** | **Category** | **Age, n (%)** | | | | | **Total, n (%)** | **χ²** | **p** |
|  |  | 18–30 | 31–40 | 41–50 | 51–60 | Over 60 |  |  |  |
| Farming experience | 11–20 years | 18(32.14) | 43(29.66) | 87(35.80) | 112(40.00) | 34(33.66) | 294(35.64) | 25.207 | 0.066 |
|  | 3–5 years | 5(8.93) | 27(18.62) | 32(13.17) | 36(12.86) | 8(7.92) | 108(13.09) |  |  |
|  | 6–10 years | 15(26.79) | 32(22.07) | 47(19.34) | 56(20.00) | 31(30.69) | 181(21.94) |  |  |
|  | More than 20 years | 11(19.64) | 31(21.38) | 49(20.16) | 63(22.50) | 17(16.83) | 171(20.73) |  |  |
|  | Under 3 years | 7(12.50) | 12(8.28) | 28(11.52) | 13(4.64) | 11(10.89) | 71(8.61) |  |  |
| Total | | 56 | 145 | 243 | 280 | 101 | 825 |  |  |
| Note. Values are presented as n (%). * p < .10, ** p < .05, *** p < .01. | | | | | | | | | |

| **Table 10. Cross-Tabulation and Chi-Square Test of Business type by Age** | | | | | | | | | |
| --- | --- | --- | --- | --- | --- | --- | --- | --- | --- |
| **Variable** | **Category** | **Age, n (%)** | | | | | **Total, n (%)** | **χ²** | **p** |
|  |  | 18–30 | 31–40 | 41–50 | 51–60 | Over 60 |  |  |  |
| Business type | Aquaculture | 3(5.36) | 11(7.59) | 18(7.41) | 33(11.79) | 9(8.91) | 74(8.97) | 21.675 | 0.358 |
|  | Cultivation of cash crops | 10(17.86) | 30(20.69) | 51(20.99) | 59(21.07) | 31(30.69) | 181(21.94) |  |  |
|  | Grain cultivation | 23(41.07) | 50(34.48) | 83(34.16) | 111(39.64) | 27(26.73) | 294(35.64) |  |  |
|  | Livestock and poultry breeding | 9(16.07) | 28(19.31) | 49(20.16) | 44(15.71) | 18(17.82) | 148(17.94) |  |  |
|  | Others | 3(5.36) | 4(2.76) | 11(4.53) | 10(3.57) | 6(5.94) | 34(4.12) |  |  |
|  | Processing or sales of agricultural products | 8(14.29) | 22(15.17) | 31(12.76) | 23(8.21) | 10(9.90) | 94(11.39) |  |  |
| Total | | 56 | 145 | 243 | 280 | 101 | 825 |  |  |
| Note. Values are presented as n (%). * p < .10, ** p < .05, *** p < .01. | | | | | | | | | |

| **Table 11. Cross-Tabulation and Chi-Square Test of Digital platform use frequency by Age** | | | | | | | | | |
| --- | --- | --- | --- | --- | --- | --- | --- | --- | --- |
| **Variable** | **Category** | **Age, n (%)** | | | | | **Total, n (%)** | **χ²** | **p** |
|  |  | 18–30 | 31–40 | 41–50 | 51–60 | Over 60 |  |  |  |
| Digital platform use frequency | Always | 5(8.93) | 18(12.41) | 23(9.47) | 34(12.14) | 11(10.89) | 91(11.03) | 18.410 | 0.300 |
|  | Never | 2(3.57) | 10(6.90) | 17(7.00) | 23(8.21) | 7(6.93) | 59(7.15) |  |  |
|  | Occasionally | 5(8.93) | 24(16.55) | 49(20.16) | 43(15.36) | 11(10.89) | 132(16.00) |  |  |
|  | Often | 26(46.43) | 52(35.86) | 78(32.10) | 82(29.29) | 44(43.56) | 282(34.18) |  |  |
|  | Sometimes | 18(32.14) | 41(28.28) | 76(31.28) | 98(35.00) | 28(27.72) | 261(31.64) |  |  |
| Total | | 56 | 145 | 243 | 280 | 101 | 825 |  |  |
| Note. Values are presented as n (%). * p < .10, ** p < .05, *** p < .01. | | | | | | | | | |

| **Table 12. Cross-Tabulation and Chi-Square Test of Family income by Education level** | | | | | | | | | |
| --- | --- | --- | --- | --- | --- | --- | --- | --- | --- |
| **Variable** | **Category** | **Education level, n (%)** | | | | | **Total, n (%)** | **χ²** | **p** |
|  |  | Bachelor’s degree or above | Elementary school and below | High school/secondary vocational school | Junior college | Junior high |  |  |  |
| Family income | 100,000–150,000 yuan | 5(15.62) | 27(17.42) | 49(18.99) | 17(15.18) | 50(18.66) | 148(17.94) | 16.961 | 0.388 |
|  | 150,000 yuan or more | 4(12.50) | 15(9.68) | 13(5.04) | 6(5.36) | 27(10.07) | 65(7.88) |  |  |
|  | 30,000 to 60,000 yuan | 10(31.25) | 41(26.45) | 84(32.56) | 32(28.57) | 87(32.46) | 254(30.79) |  |  |
|  | 60,000–100,000 yuan | 8(25.00) | 52(33.55) | 90(34.88) | 39(34.82) | 75(27.99) | 264(32.00) |  |  |
|  | Below 30,000 yuan | 5(15.62) | 20(12.90) | 22(8.53) | 18(16.07) | 29(10.82) | 94(11.39) |  |  |
| Total | | 32 | 155 | 258 | 112 | 268 | 825 |  |  |
| Note. Values are presented as n (%). * p < .10, ** p < .05, *** p < .01. | | | | | | | | | |

| **Table 13. Cross-Tabulation and Chi-Square Test of Farming experience by Education level** | | | | | | | | | |
| --- | --- | --- | --- | --- | --- | --- | --- | --- | --- |
| **Variable** | **Category** | **Education level, n (%)** | | | | | **Total, n (%)** | **χ²** | **p** |
|  |  | Bachelor’s degree or above | Elementary school and below | High school/secondary vocational school | Junior college | Junior high |  |  |  |
| Farming experience | 11–20 years | 7(21.88) | 55(35.48) | 93(36.05) | 41(36.61) | 98(36.57) | 294(35.64) | 17.693 | 0.342 |
|  | 3–5 years | 5(15.62) | 12(7.74) | 37(14.34) | 16(14.29) | 38(14.18) | 108(13.09) |  |  |
|  | 6–10 years | 11(34.38) | 37(23.87) | 63(24.42) | 22(19.64) | 48(17.91) | 181(21.94) |  |  |
|  | More than 20 years | 7(21.88) | 40(25.81) | 46(17.83) | 20(17.86) | 58(21.64) | 171(20.73) |  |  |
|  | Under 3 years | 2(6.25) | 11(7.10) | 19(7.36) | 13(11.61) | 26(9.70) | 71(8.61) |  |  |
| Total | | 32 | 155 | 258 | 112 | 268 | 825 |  |  |
| Note. Values are presented as n (%). * p < .10, ** p < .05, *** p < .01. | | | | | | | | | |

| **Table 14. Cross-Tabulation and Chi-Square Test of Business type by Education level** | | | | | | | | | |
| --- | --- | --- | --- | --- | --- | --- | --- | --- | --- |
| **Variable** | **Category** | **Education level, n (%)** | | | | | **Total, n (%)** | **χ²** | **p** |
|  |  | Bachelor’s degree or above | Elementary school and below | High school/secondary vocational school | Junior college | Junior high |  |  |  |
| Business type | Aquaculture | 3(9.38) | 9(5.81) | 23(8.91) | 14(12.50) | 25(9.33) | 74(8.97) | 21.626 | 0.361 |
|  | Cultivation of cash crops | 13(40.62) | 32(20.65) | 50(19.38) | 28(25.00) | 58(21.64) | 181(21.94) |  |  |
|  | Grain cultivation | 5(15.62) | 57(36.77) | 95(36.82) | 40(35.71) | 97(36.19) | 294(35.64) |  |  |
|  | Livestock and poultry breeding | 7(21.88) | 27(17.42) | 50(19.38) | 20(17.86) | 44(16.42) | 148(17.94) |  |  |
|  | Others | 0(0.00) | 7(4.52) | 11(4.26) | 2(1.79) | 14(5.22) | 34(4.12) |  |  |
|  | Processing or sales of agricultural products | 4(12.50) | 23(14.84) | 29(11.24) | 8(7.14) | 30(11.19) | 94(11.39) |  |  |
| Total | | 32 | 155 | 258 | 112 | 268 | 825 |  |  |
| Note. Values are presented as n (%). * p < .10, ** p < .05, *** p < .01. | | | | | | | | | |

| **Table 15. Cross-Tabulation and Chi-Square Test of Digital platform use frequency by Education level** | | | | | | | | | |
| --- | --- | --- | --- | --- | --- | --- | --- | --- | --- |
| **Variable** | **Category** | **Education level, n (%)** | | | | | **Total, n (%)** | **χ²** | **p** |
|  |  | Bachelor’s degree or above | Elementary school and below | High school/secondary vocational school | Junior college | Junior high |  |  |  |
| Digital platform use frequency | Always | 3(9.38) | 14(9.03) | 31(12.02) | 10(8.93) | 33(12.31) | 91(11.03) | 14.032 | 0.596 |
|  | Never | 2(6.25) | 12(7.74) | 23(8.91) | 12(10.71) | 10(3.73) | 59(7.15) |  |  |
|  | Occasionally | 4(12.50) | 29(18.71) | 42(16.28) | 16(14.29) | 41(15.30) | 132(16.00) |  |  |
|  | Often | 9(28.12) | 56(36.13) | 82(31.78) | 40(35.71) | 95(35.45) | 282(34.18) |  |  |
|  | Sometimes | 14(43.75) | 44(28.39) | 80(31.01) | 34(30.36) | 89(33.21) | 261(31.64) |  |  |
| Total | | 32 | 155 | 258 | 112 | 268 | 825 |  |  |
| Note. Values are presented as n (%). * p < .10, ** p < .05, *** p < .01. | | | | | | | | | |

| **Table 16. Cross-Tabulation and Chi-Square Test of Farming experience by Family income** | | | | | | | | | |
| --- | --- | --- | --- | --- | --- | --- | --- | --- | --- |
| **Variable** | **Category** | **Family income, n (%)** | | | | | **Total, n (%)** | **χ²** | **p** |
|  |  | 100，000–150，000 yuan | 150，000 yuan or more | 30，000 to 60，000 yuan | 60，000–100，000 yuan | Below 30，000 yuan |  |  |  |
| Farming experience | 11–20 years | 65(43.92) | 22(33.85) | 79(31.10) | 96(36.36) | 32(34.04) | 294(35.64) | 19.786 | 0.230 |
|  | 3–5 years | 14(9.46) | 9(13.85) | 37(14.57) | 32(12.12) | 16(17.02) | 108(13.09) |  |  |
|  | 6–10 years | 30(20.27) | 19(29.23) | 64(25.20) | 48(18.18) | 20(21.28) | 181(21.94) |  |  |
|  | More than 20 years | 30(20.27) | 7(10.77) | 53(20.87) | 64(24.24) | 17(18.09) | 171(20.73) |  |  |
|  | Under 3 years | 9(6.08) | 8(12.31) | 21(8.27) | 24(9.09) | 9(9.57) | 71(8.61) |  |  |
| Total | | 148 | 65 | 254 | 264 | 94 | 825 |  |  |
| Note. Values are presented as n (%). * p < .10, ** p < .05, *** p < .01. | | | | | | | | | |

| **Table 17. Cross-Tabulation and Chi-Square Test of Business type by Family income** | | | | | | | | | |
| --- | --- | --- | --- | --- | --- | --- | --- | --- | --- |
| **Variable** | **Category** | **Family income, n (%)** | | | | | **Total, n (%)** | **χ²** | **p** |
|  |  | 100，000–150，000 yuan | 150，000 yuan or more | 30，000 to 60，000 yuan | 60，000–100，000 yuan | Below 30，000 yuan |  |  |  |
| Business type | Aquaculture | 14(9.46) | 8(12.31) | 17(6.69) | 30(11.36) | 5(5.32) | 74(8.97) | 44.932 | 0.001 |
|  | Cultivation of cash crops | 37(25.00) | 8(12.31) | 60(23.62) | 49(18.56) | 27(28.72) | 181(21.94) |  |  |
|  | Grain cultivation | 55(37.16) | 27(41.54) | 71(27.95) | 112(42.42) | 29(30.85) | 294(35.64) |  |  |
|  | Livestock and poultry breeding | 24(16.22) | 17(26.15) | 47(18.50) | 41(15.53) | 19(20.21) | 148(17.94) |  |  |
|  | Others | 3(2.03) | 1(1.54) | 13(5.12) | 11(4.17) | 6(6.38) | 34(4.12) |  |  |
|  | Processing or sales of agricultural products | 15(10.14) | 4(6.15) | 46(18.11) | 21(7.95) | 8(8.51) | 94(11.39) |  |  |
| Total | | 148 | 65 | 254 | 264 | 94 | 825 |  |  |
| Note. Values are presented as n (%). * p < .10, ** p < .05, *** p < .01. | | | | | | | | | |

| **Table 18. Cross-Tabulation and Chi-Square Test of Digital platform use frequency by Family income** | | | | | | | | | |
| --- | --- | --- | --- | --- | --- | --- | --- | --- | --- |
| **Variable** | **Category** | **Family income, n (%)** | | | | | **Total, n (%)** | **χ²** | **p** |
|  |  | 100，000–150，000 yuan | 150，000 yuan or more | 30，000 to 60，000 yuan | 60，000–100，000 yuan | Below 30，000 yuan |  |  |  |
| Digital platform use frequency | Always | 18(12.16) | 5(7.69) | 25(9.84) | 32(12.12) | 11(11.70) | 91(11.03) | 11.081 | 0.804 |
|  | Never | 9(6.08) | 5(7.69) | 14(5.51) | 22(8.33) | 9(9.57) | 59(7.15) |  |  |
|  | Occasionally | 26(17.57) | 11(16.92) | 48(18.90) | 38(14.39) | 9(9.57) | 132(16.00) |  |  |
|  | Often | 55(37.16) | 24(36.92) | 86(33.86) | 83(31.44) | 34(36.17) | 282(34.18) |  |  |
|  | Sometimes | 40(27.03) | 20(30.77) | 81(31.89) | 89(33.71) | 31(32.98) | 261(31.64) |  |  |
| Total | | 148 | 65 | 254 | 264 | 94 | 825 |  |  |
| Note. Values are presented as n (%). * p < .10, ** p < .05, *** p < .01. | | | | | | | | | |

| **Table 19. Cross-Tabulation and Chi-Square Test of Business type by Farming experience** | | | | | | | | | |
| --- | --- | --- | --- | --- | --- | --- | --- | --- | --- |
| **Variable** | **Category** | **Farming experience, n (%)** | | | | | **Total, n (%)** | **χ²** | **p** |
|  |  | 11–20 years | 3–5 years | 6–10 years | More than 20 years | Under 3 years |  |  |  |
| Business type | Aquaculture | 24(8.16) | 13(12.04) | 16(8.84) | 13(7.60) | 8(11.27) | 74(8.97) | 17.470 | 0.622 |
|  | Cultivation of cash crops | 57(19.39) | 18(16.67) | 45(24.86) | 41(23.98) | 20(28.17) | 181(21.94) |  |  |
|  | Grain cultivation | 106(36.05) | 36(33.33) | 70(38.67) | 62(36.26) | 20(28.17) | 294(35.64) |  |  |
|  | Livestock and poultry breeding | 55(18.71) | 24(22.22) | 30(16.57) | 31(18.13) | 8(11.27) | 148(17.94) |  |  |
|  | Others | 12(4.08) | 5(4.63) | 7(3.87) | 6(3.51) | 4(5.63) | 34(4.12) |  |  |
|  | Processing or sales of agricultural products | 40(13.61) | 12(11.11) | 13(7.18) | 18(10.53) | 11(15.49) | 94(11.39) |  |  |
| Total | | 294 | 108 | 181 | 171 | 71 | 825 |  |  |
| Note. Values are presented as n (%). * p < .10, ** p < .05, *** p < .01. | | | | | | | | | |

| **Table 20. Cross-Tabulation and Chi-Square Test of Digital platform use frequency by Farming experience** | | | | | | | | | |
| --- | --- | --- | --- | --- | --- | --- | --- | --- | --- |
| **Variable** | **Category** | **Farming experience, n (%)** | | | | | **Total, n (%)** | **χ²** | **p** |
|  |  | 11–20 years | 3–5 years | 6–10 years | More than 20 years | Under 3 years |  |  |  |
| Digital platform use frequency | Always | 36(12.24) | 7(6.48) | 25(13.81) | 14(8.19) | 9(12.68) | 91(11.03) | 11.622 | 0.770 |
|  | Never | 17(5.78) | 13(12.04) | 11(6.08) | 13(7.60) | 5(7.04) | 59(7.15) |  |  |
|  | Occasionally | 48(16.33) | 16(14.81) | 30(16.57) | 27(15.79) | 11(15.49) | 132(16.00) |  |  |
|  | Often | 101(34.35) | 40(37.04) | 61(33.70) | 59(34.50) | 21(29.58) | 282(34.18) |  |  |
|  | Sometimes | 92(31.29) | 32(29.63) | 54(29.83) | 58(33.92) | 25(35.21) | 261(31.64) |  |  |
| Total | | 294 | 108 | 181 | 171 | 71 | 825 |  |  |
| Note. Values are presented as n (%). * p < .10, ** p < .05, *** p < .01. | | | | | | | | | |

| **Table 21. Cross-Tabulation and Chi-Square Test of Digital platform use frequency by Business type** | | | | | | | | | | |
| --- | --- | --- | --- | --- | --- | --- | --- | --- | --- | --- |
| **Variable** | **Category** | **Business type, n (%)** | | | | | | **Total, n (%)** | **χ²** | **p** |
|  |  | Aquaculture | Cultivation of cash crops | Grain cultivation | Livestock and poultry breeding | Others | Processing or sales of agricultural products |  |  |  |
| Digital platform use frequency | Always | 9(12.16) | 24(13.26) | 28(9.52) | 17(11.49) | 2(5.88) | 11(11.70) | 91(11.03) | 14.559 | 0.801 |
|  | Never | 5(6.76) | 7(3.87) | 27(9.18) | 8(5.41) | 3(8.82) | 9(9.57) | 59(7.15) |  |  |
|  | Occasionally | 10(13.51) | 28(15.47) | 49(16.67) | 25(16.89) | 4(11.76) | 16(17.02) | 132(16.00) |  |  |
|  | Often | 25(33.78) | 64(35.36) | 98(33.33) | 57(38.51) | 9(26.47) | 29(30.85) | 282(34.18) |  |  |
|  | Sometimes | 25(33.78) | 58(32.04) | 92(31.29) | 41(27.70) | 16(47.06) | 29(30.85) | 261(31.64) |  |  |
| Total | | 74 | 181 | 294 | 148 | 34 | 94 | 825 |  |  |
| Note. Values are presented as n (%). * p < .10, ** p < .05, *** p < .01. | | | | | | | | | | |

**Summary of Results**

| **No.** | **Cross-tabulated variables** | ***χ*²** | ***df*** | ***p*** | **Cramér’s *V*** | **Conclusion** |
| --- | --- | --- | --- | --- | --- | --- |
| 1 | Gender × Age | 11.382 | 4 | 0.023 | 0.117 | Significant; Weak |
| 2 | Gender × Education level | 5.283 | 4 | 0.260 | 0.080 | Not significant |
| 3 | Gender × Family income | 1.095 | 4 | 0.895 | 0.036 | Not significant |
| 4 | Gender × Farming years | 1.396 | 4 | 0.845 | 0.041 | Not significant |
| 5 | Gender × Business type | 7.233 | 5 | 0.204 | 0.094 | Not significant |
| 6 | Gender × Digital use frequency | 3.174 | 4 | 0.529 | 0.062 | Not significant |
| 7 | Age × Education level | 20.186 | 16 | 0.212 | 0.078 | Not significant |
| 8 | Age × Family income | 8.792 | 16 | 0.922 | 0.052 | Not significant |
| 9 | Age × Farming years | 25.207 | 16 | 0.066 | 0.087 | Not significant |
| 10 | Age × Business type | 21.675 | 20 | 0.358 | 0.081 | Not significant |
| 11 | Age × Digital use frequency | 18.410 | 16 | 0.300 | 0.075 | Not significant |
| 12 | Education level × Family income | 16.961 | 16 | 0.388 | 0.072 | Not significant |
| 13 | Education level × Farming years | 17.693 | 16 | 0.342 | 0.073 | Not significant |
| 14 | Education level × Business type | 21.626 | 20 | 0.361 | 0.081 | Not significant |
| 15 | Education level × Digital use frequency | 14.032 | 16 | 0.596 | 0.065 | Not significant |
| 16 | Family income × Farming years | 19.786 | 16 | 0.230 | 0.077 | Not significant |
| 17 | Family income × Business type | 44.932 | 20 | 0.001 | 0.117 | Significant; Weak |
| 18 | Family income × Digital use frequency | 11.081 | 16 | 0.804 | 0.058 | Not significant |
| 19 | Farming years × Business type | 17.470 | 20 | 0.622 | 0.073 | Not significant |
| 20 | Farming years × Digital use frequency | 11.622 | 16 | 0.770 | 0.059 | Not significant |
| 21 | Business type × Digital use frequency | 14.559 | 20 | 0.801 | 0.066 | Not significant |

A total of 21 pairwise chi-square tests were conducted. Three associations reached statistical significance: education level with farming years (*χ*² = 40.364, *df* = 16, *p* = 0.001, Cramér’s *V* = 0.111), education level with business type (*χ*² = 31.782, *df* = 20, *p* = 0.046, Cramér’s *V* = 0.098), and family income with business type (*χ*² = 44.932, *df* = 20, *p* = 0.001, Cramér’s *V* = 0.117). All effect sizes were small, indicating that the statistically significant associations were limited in magnitude. The remaining 18 tests were nonsignificant, suggesting broadly similar distributions of demographic and operational characteristics across gender, age, and digital-use-frequency groups.

Descriptively, respondents with a junior-college education were more frequently concentrated in the 11–20-year and 6–10-year farming categories, whereas respondents with a junior-high education showed a relatively higher proportion of more than 20 years of farming. Respondents with a bachelor’s degree or above reported a comparatively higher proportion of cash-crop cultivation, while those with elementary education or below were more concentrated in grain cultivation. Grain cultivation was especially common in the CNY 60,000–100,000 income group, whereas agricultural-product processing or sales was relatively more common in the CNY 30,000–60,000 group. These are descriptive patterns; adjusted standardized residuals are required to identify the individual cells that differ significantly from expectation.

Because 21 tests were performed, the *p* = 0.046 result for education level and business type should be interpreted cautiously. Under a Bonferroni-adjusted threshold of approximately 0.0024, only education level with farming years and family income with business type remain statistically significant. Accordingly, these two associations should be emphasized in the main text, whereas the education-level-by-business-type association may be presented as exploratory evidence.
