# Supplementary material for: Factors associated with farmers’ well-being in meaningful environments: a chain psychological association of digital literacy, psychological empowerment, and farmers’ decision-making behaviors
Source: Front Psychol. 2026 Jul 28;17:1891582. doi: 10.3389/fpsyg.2026.1891582 (PMC13457035; doi:10.3389/fpsyg.2026.1891582)
Supplement: Supplementary file 1 [file Data_Sheet_1.ZIP › Supplementary_Material_1_Questionnaire.docx]

**Factors Associated with Farmers’ Well-being in Meaningful Environments: A Chain Psychological Association of Digital Literacy, Psychological Empowerment, and Farmers’ Decision-Making Behaviors**

Questionnaire Description

Dear interviewee: Hello! This questionnaire aims to understand the relationship among meaningful rural environmental perception, digital literacy, psychological empowerment, farmers’ decision-making behaviors, and their sense of well-being. The questionnaire is intended solely for academic research; all information will be anonymized and will not involve personal privacy disclosure. Please answer based on your own actual situation; there is no right or wrong answer.

Voluntary Participation and Withdrawal Right

Your participation is entirely voluntary. You may stop filling out the questionnaire at any time without giving any reason

Withdrawing will not result in any negative consequences for you

Confirmation of Consent

Please check the box below to confirm that you have read and understood all the above information and voluntarily agree to participate in this study: □ I have read and understood the informed consent, and I voluntarily agree to participate in this study

This questionnaire uses a 5-point Likert scoring system: 1 = strongly disagree, 2 = disagree, 3 = neutral, 4 = agree, 5 = strongly agree. Please check the box under the number that best fits your situation.

# Part One: Basic Information

| **Title** | **Options** |
| --- | --- |
| 1. Your gender | A. Male B. Female |
| 2. Your age | A. 18–30 B. 31–40 C. 41–50 D. 51–60 E. Over 60 |
| 3. Your level of education | A. Elementary school and below B. Junior high C. High school/secondary vocational school D. Junior college E. Bachelor’s degree or above |
| 4. Your family’s annual income level | A. Below 30,000 yuan B. 30,000–60,000 yuan C. 60,000–100,000 yuan D. 100,000–150,000 yuan E. 150，000 yuan or more |
| 5. The years you have been engaged in agricultural production | A. Under 3 years B. 3–5 years C. 6–10 years D. 11–20 years E. More than 20 years |
| 6. The type of farming you run in your family | A. Grain cultivation B. Cultivation of cash crops C. Livestock and poultry farming D. Aquaculture E. Processing or sales of agricultural products F. Others |
| 7. Do you often use your smartphone, internet, or digital platforms to obtain agriculture-related information? | A. Never B. Occasionally C. Sometimes D. Often E. Always |

# Part Two: Core Variable Scale

The following questions are scored on a scale of 1–5 points; the higher the score, the higher the level of the variable.

## (1) Meaningful Environmental Perception Scale

| **Number** | **Topic** | **1** | **2** | **3** | **4** | **5** |
| --- | --- | --- | --- | --- | --- | --- |
| X1 | I believe the current rural living environment makes me feel valued and a sense of belonging. |  |  |  |  |  |
| X2 | I can feel that agricultural production is not only a means of livelihood but also carries family, community, or social significance. |  |  |  |  |  |
| X3 | Public services, neighborhood relations, and production conditions in the village support me in achieving a better life. |  |  |  |  |  |
| X4 | I believe I can play a certain role in rural development. |  |  |  |  |  |
| X5 | The current rural digitalization, industrialization, and governance environment give me greater confidence in future life. |  |  |  |  |  |
| X6 | I was able to gain a lasting sense of meaning from agricultural labor and rural life. |  |  |  |  |  |

## Digital Literacy Scale (M1)

| **Number** | **Topic** | **1** | **2** | **3** | **4** | **5** |
| --- | --- | --- | --- | --- | --- | --- |
| DL1 | I am proficient in using smartphones, WeChat, short video platforms, or agricultural information platforms. |  |  |  |  |  |
| DL2 | I can search online for information on agricultural production, market prices, policy subsidies, and more. |  |  |  |  |  |
| DL3 | I was able to judge whether online agricultural information was reliable. |  |  |  |  |  |
| DL4 | I will use digital platforms to understand sales channels or market demand for agricultural products. |  |  |  |  |  |
| DL5 | I am willing to learn new digital tools to improve agricultural production or family operations. |  |  |  |  |  |
| DL6 | I was able to communicate with technicians, cooperatives, buyers, or other farmers through online channels. |  |  |  |  |  |
| DL7 | When encountering issues with digital devices or network platforms, I usually find solutions. |  |  |  |  |  |

## (3) Mental Empowerment Scale (M2)

| **Number** | **Topic** | **1** | **2** | **3** | **4** | **5** |
| --- | --- | --- | --- | --- | --- | --- |
| PE1 | I believe I have the ability to improve the family’s agricultural operations. |  |  |  |  |  |
| PE2 | I believe my judgment can help families make better production or business decisions. |  |  |  |  |  |
| PE3 | When facing agricultural risks or market changes, I am still able to respond proactively. |  |  |  |  |  |
| PE4 | I feel that I have a certain degree of initiative in family production and management. |  |  |  |  |  |
| PE5 | I am willing to try new agricultural techniques, business models, or sales channels. |  |  |  |  |  |
| PE6 | I believe my efforts can have a positive impact on family income and quality of life. |  |  |  |  |  |
| PE7 | Even when facing difficulties, I believe I can find a solution. |  |  |  |  |  |

## (4) Farmers’ Decision-Making Behavior Scale (M3)

| **Number** | **Topic** | **1** | **2** | **3** | **4** | **5** |
| --- | --- | --- | --- | --- | --- | --- |
| DB1 | Before making agricultural production decisions, I proactively gather market, technical, or policy information. |  |  |  |  |  |
| DB2 | I adjust planting, breeding, or sales arrangements according to market price changes. |  |  |  |  |  |
| DB3 | I am willing to adopt new agricultural technologies, machinery, or digital management methods. |  |  |  |  |  |
| DB4 | I compare different sales channels before deciding on how to sell agricultural products. |  |  |  |  |  |
| DB5 | I will make more rational business choices based on family resources, risks, and returns. |  |  |  |  |  |
| DB6 | I am willing to participate in new forms of agricultural organization such as cooperatives, e-commerce platforms, or collective operations. |  |  |  |  |  |
| DB7 | When facing uncertainty, I reduce decision-making risk through consulting, learning, or experimentation. |  |  |  |  |  |

## (5) Farmers’ Happiness Scale

| **Number** | **Topic** | **1** | **2** | **3** | **4** | **5** |
| --- | --- | --- | --- | --- | --- | --- |
| Y1 | Overall, I am satisfied with my current life. |  |  |  |  |  |
| Y2 | I feel that my family life is relatively stable and gives me a sense of security. |  |  |  |  |  |
| Y3 | I often find fulfillment in agricultural production or rural life. |  |  |  |  |  |
| Y4 | I am confident in the future improvement of my family’s income and living standards. |  |  |  |  |  |
| Y5 | I feel that my current life is hopeful. |  |  |  |  |  |
| Y6 | My relationships with family, neighbors, or village members make me happy. |  |  |  |  |  |
| Y7 | Even when life is stressful, I can still feel positive emotions. |  |  |  |  |  |
| Y8 | Compared to the past, I believe my quality of life has improved. |  |  |  |  |  |
